# A conserved mitochondrial surveillance pathway is required for defense against *Pseudomonas aeruginosa*

**DOI:** 10.1371/journal.pgen.1006876

**Published:** 2017-06-29

**Authors:** Elissa Tjahjono, Natalia V. Kirienko

**Affiliations:** Department of BioSciences, Rice University, Houston, Texas, United States of America; The University of Texas Health Science Center at Houston, UNITED STATES

## Abstract

All living organisms exist in a precarious state of homeostasis that requires constant maintenance. A wide variety of stresses, including hypoxia, heat, and infection by pathogens perpetually threaten to imbalance this state. Organisms use a battery of defenses to mitigate damage and restore normal function. Previously, we described a *Caenorhabditis elegans-Pseudomonas aeruginosa* assay (Liquid Killing) in which toxicity to the host is dependent upon the secreted bacterial siderophore pyoverdine. Although pyoverdine is also indispensable for virulence in mammals, its cytological effects are unclear. We used genetics, transcriptomics, and a variety of pathogen and chemical exposure assays to study the interactions between *P*. *aeruginosa* and *C*. *elegans*. Although *P*. *aeruginosa* can kill *C*. *elegans* through at least 5 different mechanisms, the defense responses activated by Liquid Killing are specific and selective and have little in common with innate defense mechanisms against intestinal colonization. Intriguingly, the defense response utilizes the phylogenetically-conserved ESRE (Ethanol and Stress Response Element) network, which we and others have previously shown to mitigate damage from a variety of abiotic stresses. This is the first report of this networks involvement in innate immunity, and indicates that host innate immune responses overlap with responses to abiotic stresses. The upregulation of the ESRE network in *C*. *elegans* is mediated in part by a family of bZIP proteins (including ZIP-2, ZIP-4, CEBP-1, and CEBP-2) that have overlapping and unique functions. Our data convincingly show that, following exposure to *P*. *aeruginosa*, the ESRE defense network is activated by mitochondrial damage, and that mitochondrial damage also leads to ESRE activation in mammals. This establishes a role for ESRE in a phylogenetically-conserved mitochondrial surveillance system important for stress response and innate immunity.

## Author summary

It is increasingly clear that living organisms surveil their cellular structures and physiology to gain early warning about the presence and activity of pathogens or abiotic threats (like hypoxia, hyper- or hypothermia, oxidative damage, etc.). Upon recognizing dysfunction, host defense networks are engaged to prevent and mitigate the damage. Here we report the involvement of a phylogenetically-conserved defense network, called ESRE, in mitochondrial surveillance in *C*. *elegans* and mammals. Our report expands the function of this network to include innate immunity in addition to its role in abiotic stress response. We also observed that a variety of mitochondrial poisons, and the opportunistic human pathogen *Pseudomonas aeruginosa*, activate the ESRE network, suggesting that mitochondrial surveillance is a key facet of its regulation. Since mitochondrial health is an important factor in many diseases, having a better understanding of how cells perceive and respond to damage to this organelle is critical for understanding numerous aspects of human health.

## Introduction

All living organisms encounter unfavorable stimuli, including both abiotic stressors and pathogens that interfere with their fitness and survival. To recover their homeostatic balance, they activate conserved defense networks. These include classical immune (innate and adaptive) pathways to respond to pathogens and other defenses to mitigate damage from heat shock, hypoxia, DNA damage, and unfolded protein responses in the endoplasmic reticulum (ER) and mitochondria. Each pathway triggers the production of a myriad of effectors to minimize further damage, mitigate and repair existing damage, and restore organismal health.

Intensive study has begun to shed light on many host defense pathways, helping to identify both the signals detected and the master transcriptional regulators that coordinate the defense response. For example, most organisms use pattern recognition receptors like the well-known Toll-Like Receptor family to detect a variety of structural determinants (such as lipopolysaccharide, flagellin, dsRNA, etc., commonly known as 'Pathogen-Associated Molecular Patterns', or PAMPS) shared by large numbers of pathogens [1]. Pattern recognition receptors are also triggered by normal biomolecules out of their proper context (e.g., extracellular ATP is recognized as a danger signal, as it typically indicates the lysis of nearby cells [2–4]). A wide variety of ligands, including ATP, uric acid, DNA, dsRNA, and misfolded proteins have been identified to act in this fashion [5–7], and are generally referred to as 'Damage-Associated Molecular Patterns', or DAMPs.

DAMPs are also released when organelles are damaged. For example, mitochondria release N-formylated peptides and mitochondrial DNA into the cytosol, which are recognized by the formyl peptide receptor and TLR9, respectively [8–11]. In this fashion, DAMPs serve an important role in organellar surveillance. These programs can monitor organelle function and integrity, as shown by the unfolded protein responses of the ER and mitochondria or mitochondrial dynamics that maintain homeostasis. It is thought that surveillance systems of this type may have developed as an early warning system to identify surreptitious pathogen activity [12], but these surveillance programs also provide invaluable feedback when normal metabolism has gone awry.

*C*. *elegans* is widely used as a model for investigating host defenses and surveillance programs [12–19]. We developed a new, liquid-based assay using *C*. *elegans* to facilitate the identification of novel treatments for the opportunistic human pathogen *Pseudomonas aeruginosa* [20–23]. Characterization of the assay demonstrated that pathogenesis strongly depended upon production of the bacterial siderophore pyoverdine [24,25], which is indispensable for virulence in several mammalian infection models [26–28]. We showed that pyoverdine damages mitochondria, targeting them for mitophagic turnover, and activating a hypoxia-like defense response; similar phenomena were observed when worms were exposed to the chemical chelator 1,10-phenanthroline [24,29].

In this study, we aimed to elucidate the defense mechanisms used by *C*. *elegans* to respond to pyoverdine exposure. We determined that the transcriptional response to pyoverdine is very different from classical, intestinal colonization-related immune responses in *C*. *elegans*. Moreover, we show that the conserved *E*thanol and *S*tress *R*esponse *E*lement (ESRE) master stress response network is vital for defense against pyoverdine, and that a family of bZIP transcription factors mediate this activity. Finally, we show that disrupting mitochondrial function in *C*. *elegans* and in mammals activates the ESRE network, demonstrating that it plays a key role in surveillance immunity, specifically by assessing mitochondrial health.

## Results

### *C*. *elegans* response to *P*. *aeruginosa* is contingent upon context of exposure

*P*. *aeruginosa* uses a remarkable variety of virulence factors to colonize and infect many hosts [30–32]. Even within a single host, a myriad of virulence factors is often utilized, including quorum sensing, biofilms, and a variety of toxins [33–35]. At least five different *P*. *aeruginosa-C*. *elegans* pathosystems have been described [36], including our recently developed liquid-based model (referred to as 'Liquid Killing') that requires the siderophore pyoverdine [23,24]. Pyoverdine damages host mitochondria (triggering mitophagy in the process) and induces a hypoxic response [24,29]. It is worth noting that the involvement of pyoverdine in Liquid Killing makes it unique amongst *P*. *aeruginosa* invertebrate pathogenesis models. The differences between this pathosystem and the others make it an excellent model to continue the process of elucidating whether the host detects the presence of a pathogen using PAMPS or DAMPS.

**Fig 1:**
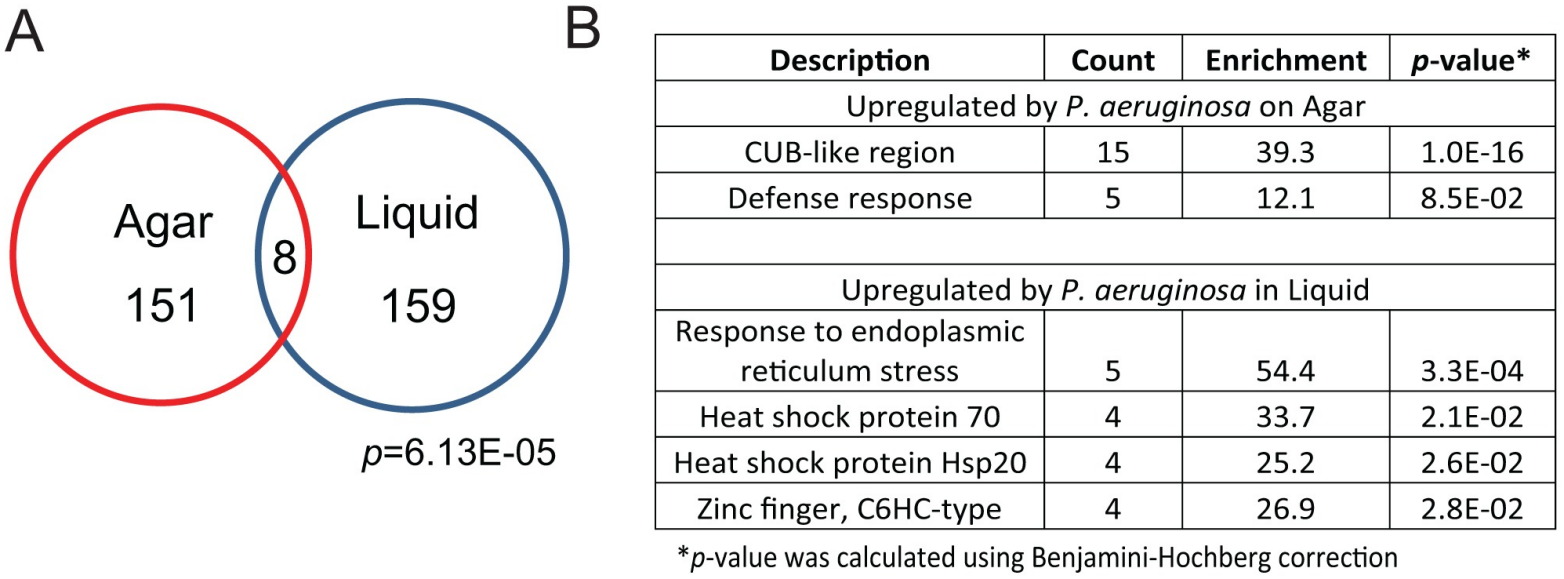
Host defense against *P*. *aeruginosa*-mediated killing in liquid is mediated via different factors than infection on plates. **(A)** Venn diagram of genes upregulated in Slow Killing (i.e., agar-based infection) and Liquid Killing (i.e., siderophore mediated intoxication). The number of genes in each category is presented and *p*-values (based on the hypergeometric distribution) are shown. **(B)** Lists of Gene Ontology (GO) groups overrepresented in the transcriptional responses against *P*. *aeruginosa* in Slow Killing (top) or Liquid Killing (bottom).

To address this question, we performed microarray-based transcriptional profiling of *C*. *elegans glp-4(bn2)*, exposed to *P*. *aeruginosa* PA14 in liquid (Liquid Killing) and on agar plates (Slow Killing) as well as corresponding *E*. *coli* controls (see S1 Table and Methods for details). Microarray analysis correlated well with qRT-PCR results (S1 Fig). As the Liquid Killing assay uses conditionally sterile *glp-4(bn2)* worms, we verified that *glp-4(bn2)* and wild-type cohorts showed similar transcriptional profiles (S2 Fig). Of the 159 genes upregulated under Liquid Killing conditions, only 8 were also upregulated under Slow Killing conditions (Fig 1A, *p* = 6.1E-05, see also S2 Table for a list of the genes). Although the overlap is statistically significant, it is likely that the biological significance is limited. For example, Slow Killing (like most other agar-based infections in *C*. *elegans*) strongly relies on activation of the PMK-1/p38 MAPK pathway for defense [37–39], while activation of this same pathway is detrimental in Liquid Killing [29]. Correspondingly, gene ontology (GO) enrichment for upregulated genes during Slow Killing predominantly involved genes with CUB-like regions and defense response categories (similar to what was published by Troemel and colleagues [39], while Liquid Killing was dominated by genes involved in endoplasmic reticulum stress and small heat shock proteins (Fig 1B).

**Table 1:** Host response to *P*. *aeruginosa* is dependent on pathogen virulence.

Condition 1	# Genes	Condition 2	# Genes	Overlap	*p*-value
Slow Killing	159	PMK-1 Dependent (PMID: 17096597)	101	11	10^−10^
Liquid Killing	167			2	0.21
Slow Killing	159	ZIP-2 Dependent (PMID: 20133860)	25	4	10^−5^
Liquid Killing	167			15	10^−25^
Slow Killing	159	Hypoxia Responsive (PMID: 15781453)	110	2	0.22
Liquid Killing	167			22	10^−24^
Slow Killing	159	*S*. *aureus* Infection (PMID: 20617181)	189	42	10^−49^
Liquid Killing	167			4	0.077
Slow Killing	159	*Y*. *pestis* Infection (PMID: 20133945)	114	16	10^−16^
Liquid Killing	167			0	1

We compared genes upregulated under Liquid Killing to similar lists from other infection conditions (including *P*. *aeruginosa* on solid media, *Y*. *pestis*, and *S*. *aureus*). Generally, there was little overlap between Liquid Killing and other pathogens. Similarly, PMK-1-dependent genes, which are frequently responsive to pathogenic bacteria (including being enriched in our Slow Killing microarray), showed little overlap with genes upregulated during Liquid Killing (Table 1). In contrast, a significant number of both ZIP-2-dependent and hypoxic responsive genes were upregulated under Liquid Killing conditions (*p* = 10^−25^ and *p* = 10^−24^, respectively).

### Genes upregulated by Liquid Killing belong to the ESRE network

**Fig 2:**
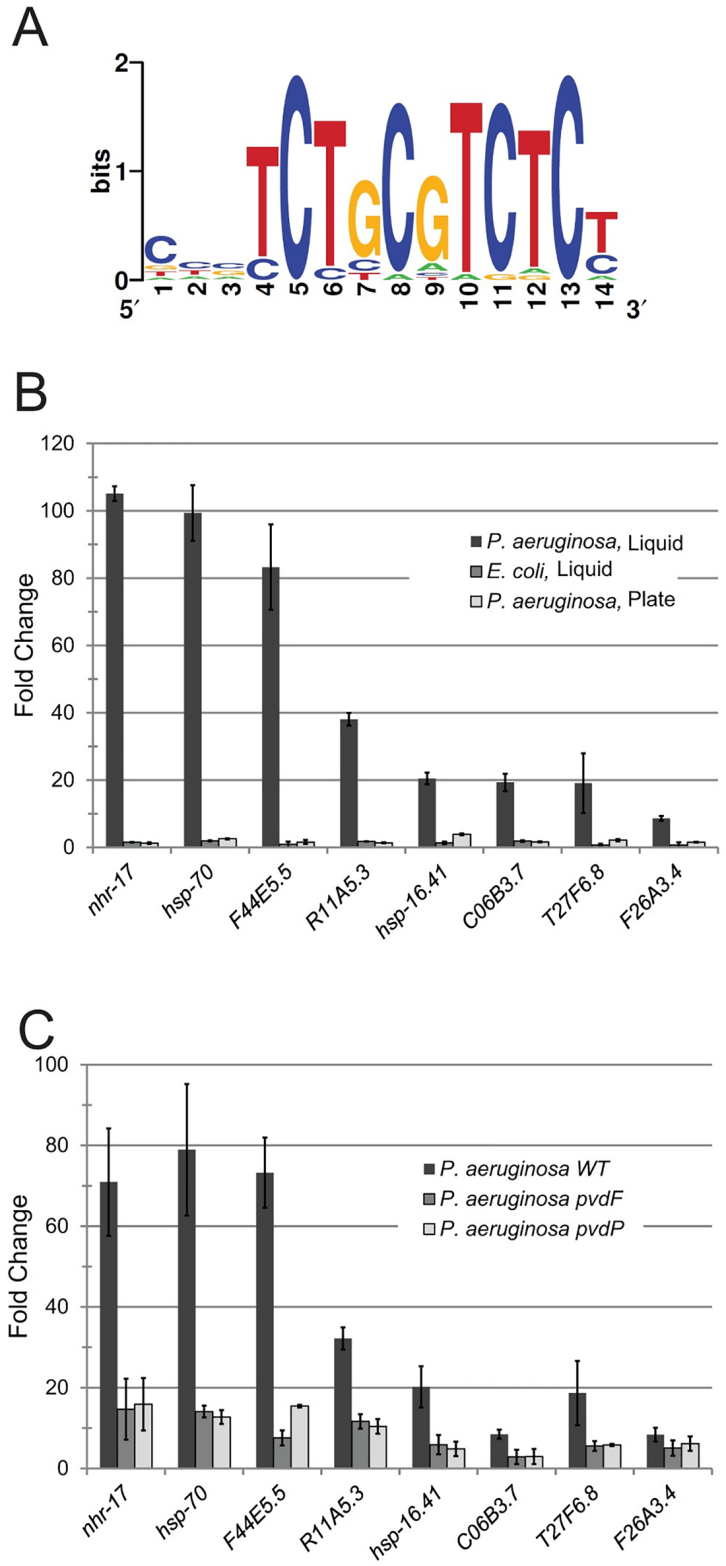
Activation of the ESRE defense network requires the presence of the siderophore pyoverdine. **(A)** Logogram of an 11-nucleotide motif identified in a cluster of genes specifically upregulated in Liquid Killing, as compared to other infectious conditions. **(B)** Expression of ESRE-containing genes in worms exposed to: *P*. *aeruginosa* in liquid, their normal (*E*. *coli*) bacterial food in liquid, or infected with *P*. *aeruginosa* on plates. Fold changes were determined by qPCR and were normalized to a cohort fed with *E*. *coli* on solid media. **(C)** Expression of ESRE genes after exposure to either wild-type *P*. *aeruginosa* or *P*. *aeruginosa* mutants with defects in pyoverdine biosynthesis. Fold changes were determined by qPCR and were normalized to a cohort exposed to *E*. *coli* under Liquid Killing conditions. Error bars represent SEM.

We were interested in identifying a transcriptome response specific to exposure to *P*. *aeruginosa* under Liquid Killing conditions, particularly in its distinction from more generic infection responses. We performed a meta-analysis of transcriptome profiles using our data for Liquid Killing and Slow Killing and published data from *C*. *elegans* exposed to other pathogens (S3A Fig). 167 genes upregulated in Liquid Killing were chosen for analysis, and normalized expression data from each transcriptional profile were used to generate clusters of genes with similar expression patterns. One cluster, containing approximately 50 genes (S3B Fig, Methods) showed upregulation only in Liquid Killing. We hypothesized that the coexpression of these genes may indicate the presence of one or more shared transcriptional regulators controlling their expression. Therefore, we searched for enrichment of nucleotide motifs that could represent potential binding sites in their promoters. Computational analysis of the upstream regions identified a strongly conserved, 11-nucleotide sequence (Fig 2A). This motif, called the ESRE [40] has been shown to work with the PBAF chromatin remodeling complex to control the response to pleiotropic abiotic stresses [40–43]. Statistical analysis demonstrated overrepresentation of this motif in the previously described cluster of genes strongly upregulated specifically in Liquid Killing (present in >40% of genes, *p* = 1.1 E-10) and in the 167 genes upregulated by Liquid Killing; but not in genes upregulated by *P*. *aeruginosa* on agar or infection with other pathogens (S3 Table). This suggested that involvement of the ESRE network was likely specific to exposure to *P*. *aeruginosa* in liquid.

We used two methods to validate the importance of the ESRE motif in the *P*. *aeruginosa* Liquid Killing assay. First, qRT-PCR was used to evaluate expression of ESRE genes in worms exposed to either *P*. *aeruginosa* on agar, *E*. *coli* in liquid, or *P*. *aeruginosa* in liquid. Significant upregulation of ESRE genes was found only in worms exposed to *P*. *aeruginosa* in liquid (Fig 2B). Second, we identified 42 ESRE genes specifically upregulated in response to Liquid Killing for which correct RNAi constructs were available [44,45] and that did not compromise growth on *E*. *coli*. These 42 genes were tested for involvement in resistance to Liquid Killing. Ten genes showed increased sensitivity in at least three out of four replicates, reinforcing the importance of the ESRE motif (S4 Fig).

### *P*. *aeruginosa*-mediated activation of the ESRE network requires pyoverdine

Liquid Killing is largely contingent upon the production of pyoverdine, as virulence is attenuated in *P*. *aeruginosa* mutants with compromised pyoverdine biosynthesis [24]. This lead to the obvious hypothesis that pyoverdine is necessary for the activation of ESRE genes. To test this, we assayed ESRE gene expression in worms exposed to *P*. *aeruginosa pvdF* and *pvdP* mutants, which are incapable of producing pyoverdine. Upregulation of ESRE genes was substantially compromised (Fig 2C), supporting the idea that the ESRE pathway responds to pyoverdine. This raised an intriguing question: was the host recognizing pyoverdine (indicating that the siderophore is a PAMP) or is the host detecting iron removal (i.e., the damage caused by the toxin, implying a DAMP-mediated response).

### Exposure to phenanthroline also induces ESRE gene expression

**Fig 3:**
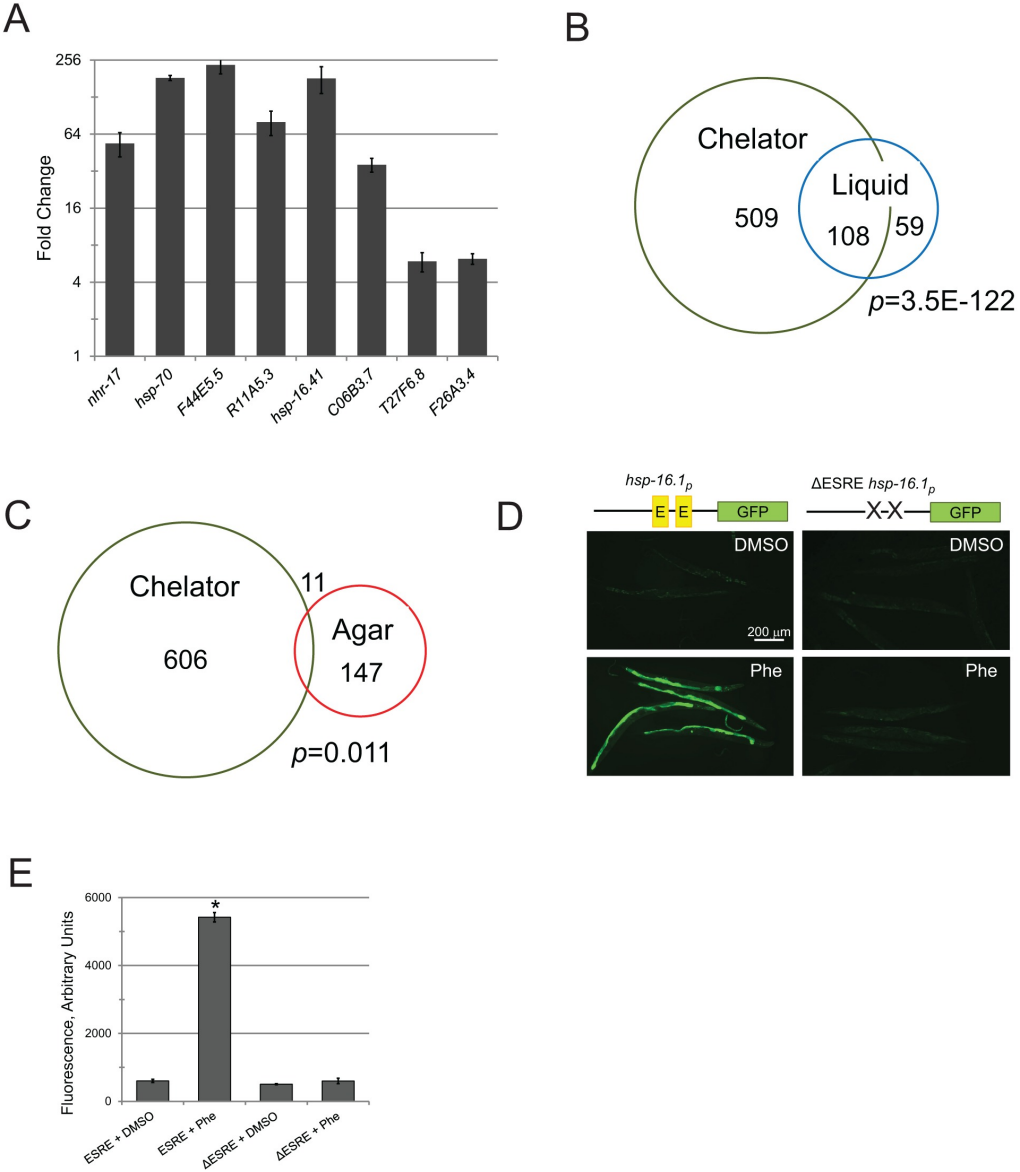
The ESRE gene network is activated by acute iron removal. **(A)** Expression of ESRE-containing genes in worms exposed to 1 mM 1,10-phenanthroline. Fold changes were normalized to vehicle (DMSO) control. **(B-C)** Venn diagrams of genes upregulated by exposure to 1 mM 1,10-phenanthroline (Chelator) and either *P*. *aeruginosa* exposure in liquid **(B)** or *P*. *aeruginosa* infection on agar plates **(C)**. In each case, the number of genes in each category is shown, as is the *p*-value (based on hypergeometric distribution). **(D)** Fluorescence microscopy of reporters with (left) or without (right) the ESRE element that were treated with 1 mM 1,10-phenanthroline (Phe) or vehicle (DMSO). Representative images are shown; three biological replicates (with ~50 worms / replicate) were analyzed. A schematic of the promoter region for the construct is shown for convenience. **(E)** Quantification of GFP fluorescence for a P_*hsp-16*.*1*_::GFP reporter with or without the ESRE element that was treated with vehicle (DMSO) or 1 mM 1,10-phenanthroline (Phe). Error bars represent SEM.

We have previously demonstrated that another chemical chelator, 1,10-phenanthroline (hereafter referred to as "phenanthroline") recapitulates many key aspects of pyoverdine intoxication, such as activation of hypoxic response, imbalance of *C*. *elegans’* mitochondrial homeostasis with subsequent fragmentation of mitochondrial network, and activation of mitophagy [24,29]. When we tested whether exposure to 1 mM phenanthroline would similarly induce ESRE gene expression, we saw strong gene upregulation (Fig 3A). As this chelator is structurally distinct from pyoverdine, it is likely that at least part of the ESRE response occurs via a DAMP/surveillance mechanism.

To widen these findings to include the entire genome, we profiled transcriptomes of worms exposed to phenanthroline (S4 Table). We saw a dramatic overlap: ~2/3 genes upregulated by *P*. *aeruginosa* in Liquid Killing were also upregulated after phenanthroline treatment (*p* = 10^−122^, Fig 3B). Interestingly, the 108 genes in this group were associated with GO categories reminiscent of stress response (S5 Table). In contrast, the 59 genes that were specific to Liquid Killing displayed enrichment in a category related to metabolism (Transferases), while the genes specifically upregulated after phenanthroline exposure exhibited a strong detoxification signature (S6 and S7 Tables). This difference could be due to the relatively higher concentration of phenanthroline (compared to pyoverdine) used, resulting in a larger number of effected genes. Alternatively, it is also possible that phenanthroline, for some reason, more effectively activates detoxification genes.

As was observed for Liquid Killing, *P*. *aeruginosa* infection on agar upregulated a disparate set of genes from phenanthroline exposure (*p* = 0.011, Fig 3C). We also examined how many genes upregulated by phenanthroline treatment were dependent upon PMK-1, ZIP-2, or were responsive to hypoxia, *S*. *aureus*, and *Y*. *pestis*. In each case, the comparison to phenanthroline was quite similar to what we observed for Liquid Killing (S8 Table). ESRE genes were also overrepresented in genes upregulated after phenanthroline treatment (14.3%, *p* = 10^−7^). These results support our previous conclusions that host response to *P*. *aeruginosa* in Liquid Killing is activated after recognition of pyoverdine-mediated damage.

Although ESRE genes were overrepresented in the response to Liquid Killing and phenanthroline, the possibilities remained that the motif was a computational artifact or that the motif was irrelevant for gene expression in this context. To rule these out, we used GFP reporter strains based on the *hsp-16*.*1* promoter [43]. The *hsp-16*.*1* promoter includes two ESRE motifs, and drives transient expression of a small heat shock protein upon exposure to various stresses [41]. Exposure of the P_*hsp-16*.*1*_::GFP reporter to phenanthroline significantly increased GFP expression (Fig 3D). Efficient expression required intact ESRE sites, as GFP expression was abolished in an otherwise identical promoter where ESRE sites were removed (Fig 3D and 3E). *P*. *aeruginosa* also activated the P_*hsp-16*.*1*_::GFP reporter (S5 Fig). Combined, these results emphasize that the ESRE element is necessary for gene activation after acute iron removal.

### Compromising mitochondrial activity induces the ESRE network in *C*. *elegans* and mammals

**Fig 4:**
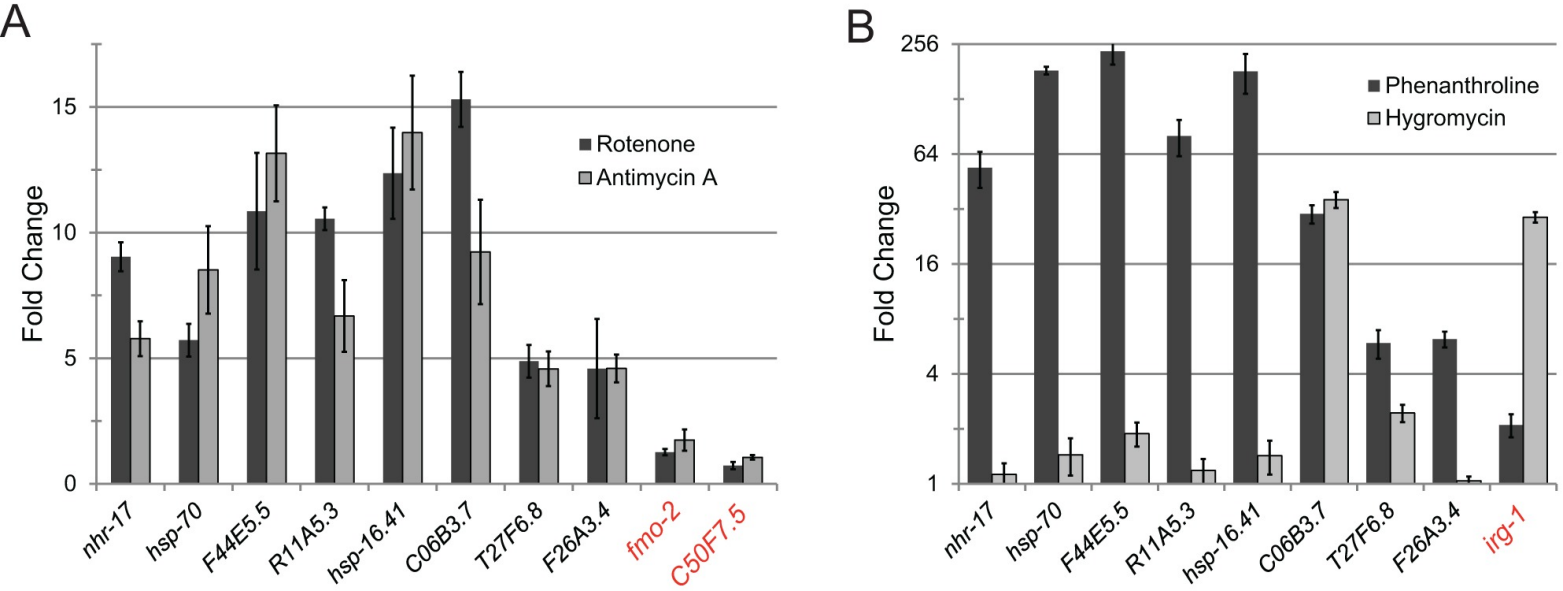
Mitochondrial disruption specifically induces the ESRE network. **(A)** Expression of ESRE-containing genes in worms exposed to 50 μM rotenone or 50 μM antimycin A. Fold changes are normalized to vehicle (DMSO) control. **(B)** Expression of ESRE-containing and non-ESRE containing genes (red label) after treatment with 1 mM 1,10-phenanthroline (Phe) or with 80 μg/mL hygromycin B (Hygro). *irg-1* is a hygromycin-responsive gene used as a control. Fold changes are normalized to vehicle (DMSO) control. Error bars represent SEM.

Despite their structurally disparate triggers, the transcriptional responses to Liquid Killing and to a small molecule iron chelator were very similar, suggesting that *C*. *elegans* may be directly monitoring intracellular iron stores. Alternatively, the removal of iron from host organelles, such as mitochondria, may disrupt their structure and/or function. We surmise that one or both of these events are recognized as a DAMP, triggering the ESRE host defense network. Therefore, we tested whether non-chelating toxins that induce mitochondrial damage activate ESRE genes’ transcription. *glp-4(bn2)* worms were exposed to either rotenone or antimycin A; both of which compromise oxidative phosphorylation, either by disrupting electron transfer from Complex I to ubiquinol or by preventing ubiquinol oxidation, respectively. Both compounds caused significant upregulation of ESRE genes (Fig 4A), indicating that compromising mitochondrial activity is sufficient to cause ESRE network induction.

The core cellular process surveillance paradigm suggests that a variety of cellular events are monitored for disruption or dysfunction, possibly as an early pathogen warning system [6,46–48]. To assess the specificity of ESRE gene upregulation, we treated worms with hygromycin, a well-known inhibitor of translation elongation. Although hygromycin caused significant upregulation of *irg-1*, an established target [49,50], ESRE genes in general showed little to no response (Fig 4B). In addition, neither tunicamycin (which induces ER stress [51]), bortezomib (a proteasome inhibitor [52]), nor ToxA (a different toxin produced by *P*. *aeruginosa* that hampers translation, but has no known effect on mitochondria [49]) caused upregulation of ESRE genes (S6 Fig). This strongly indicates that the ESRE network is specifically activated by mitochondrial damage, and is not merely a response to general disruption of cellular processes.

**Fig 5:**
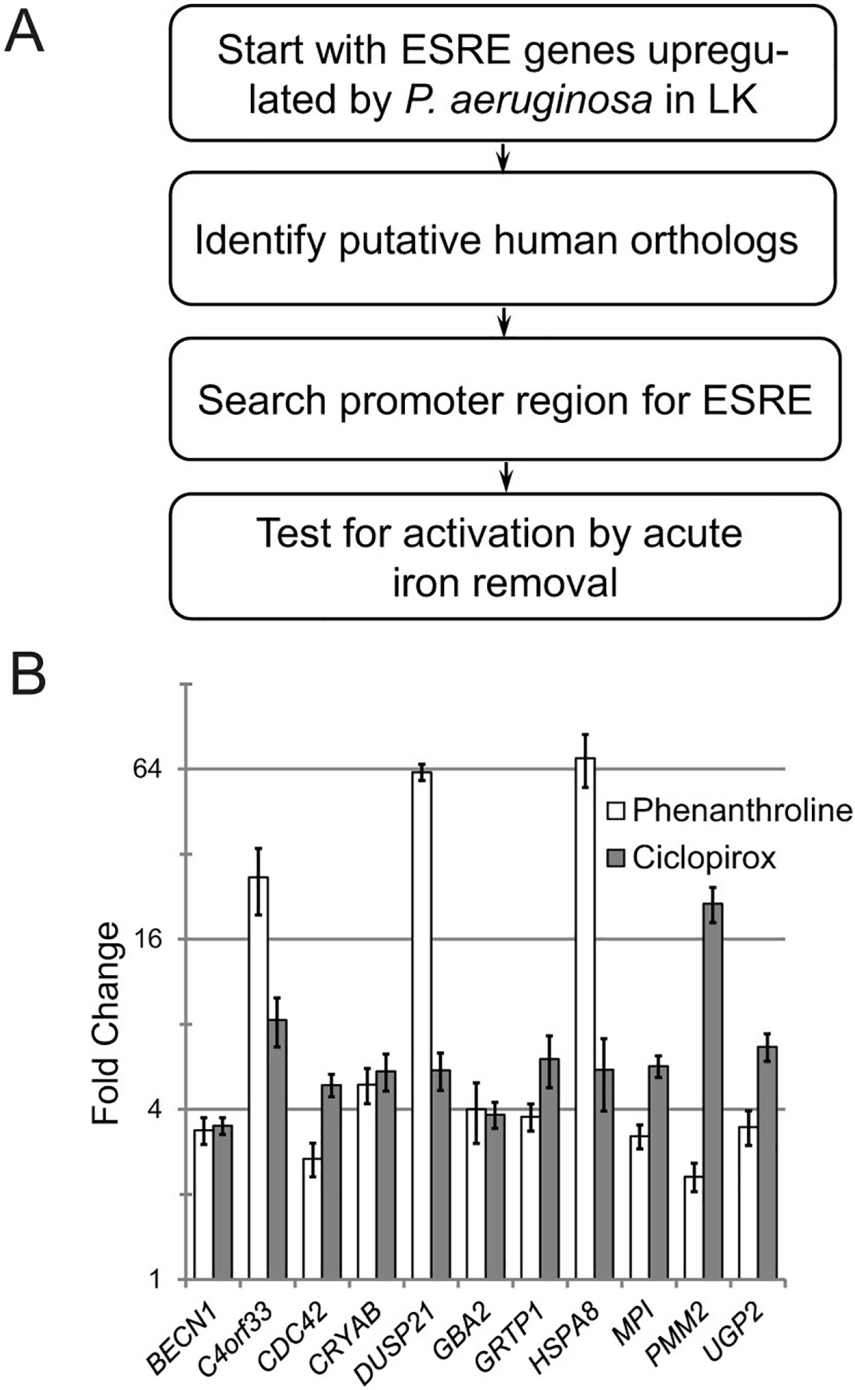
Mitochondrial surveillance and ESRE defense network activation are evolutionarily conserved. **(A)** Scheme used to identify human ESRE genes orthologous to *C*. *elegans* ESRE genes. **(B)** Expression of human ESRE genes in HEK293T cells after treatment with 0.5 mM 1,10-phenanthroline or with 0.1 mM ciclopirox olamine for 12 h. Fold changes were normalized to vehicle (DMSO) control. Error bars represent SEM.

The ESRE network is evolutionarily conserved [42,53], and chelators have been demonstrated to damage mammalian mitochondria [29,54,55]. Therefore, we were interested in determining whether synthetic chelators also activate the ESRE network in mammals, indicating a conservation of its function in mitochondrial surveillance. We used a previously described process to identify human orthologs of *C*. *elegans* ESRE genes [42] (Fig 5A). In brief, we identified putative human orthologs by using *C*. *elegans* ESRE genes that were upregulated by pyoverdine as queries for reciprocal BLAST. We verified the presence of at least one ESRE consensus motif in the promoter regions (see S9 Table for a list of selected gene pairs). For putative orthologs retaining ESRE consensus sites, we used qRT-PCR to measure gene expression in HEK293T cells treated with the iron chelating agents ciclopirox olamine or phenanthroline (Fig 5B). The compounds significantly induced expression of all 11 ESRE genes tested.

### A family of bZIP transcription factors are involved in responding to mitochondrial damage

**Fig 6:**
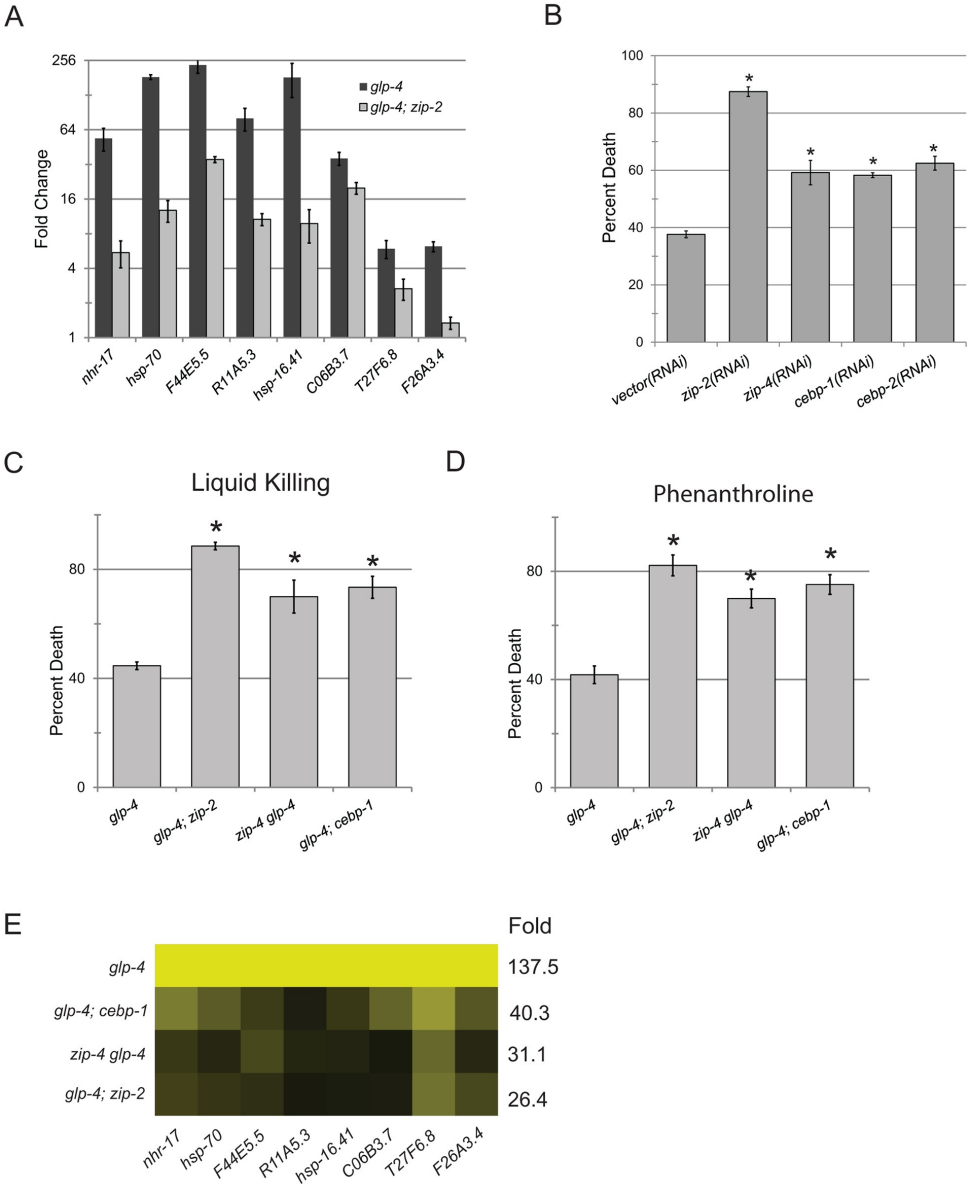
ESRE network transcription requires bZIP transcription factors. **(A)** Expression of ESRE-containing genes in *glp-4(bn2)* or *glp-4(bn2); zip-2(tm4248)* mutants treated with 1 mM 1,10-phenanthroline. Fold changes were normalized to untreated genotypic cohorts. **(B**) *glp-4(bn2)* worms were grown on a panel of RNAi strains that knock down expression of *zip-2* or one of three closely related bZIP transcription factors (*zip-4*, *cebp-1*, and *cebp-2*). Proportion of worms killed by *P*. *aeruginosa* is shown for each condition. **(C, D)** Survival of a panel of bZIP mutants exposed to Liquid Killing **(C)** or 1 mM 1,10-phenanthroline **(D)**. **(E)** Expression of ESRE-containing genes in a panel of bZIP mutants exposed to Liquid Killing. Expression for each gene was first normalized to untreated genotypic cohort and then normalized to fold changes in *glp-4(bn2)* controls. Yellow indicates WT expression level; darker shades reflect downregulation relative to wild type. Numbers in the “Fold” column show the arithmetic mean for the entire set of raw fold change for gene expression in worms exposed to Liquid Killing compared to untreated genotypic cohorts (first round of normalization). Statistical significance was determined via Student’s t-test, error bars represent SEM, asterisks represent *p*-value < 0.01.

**Fig 7:**
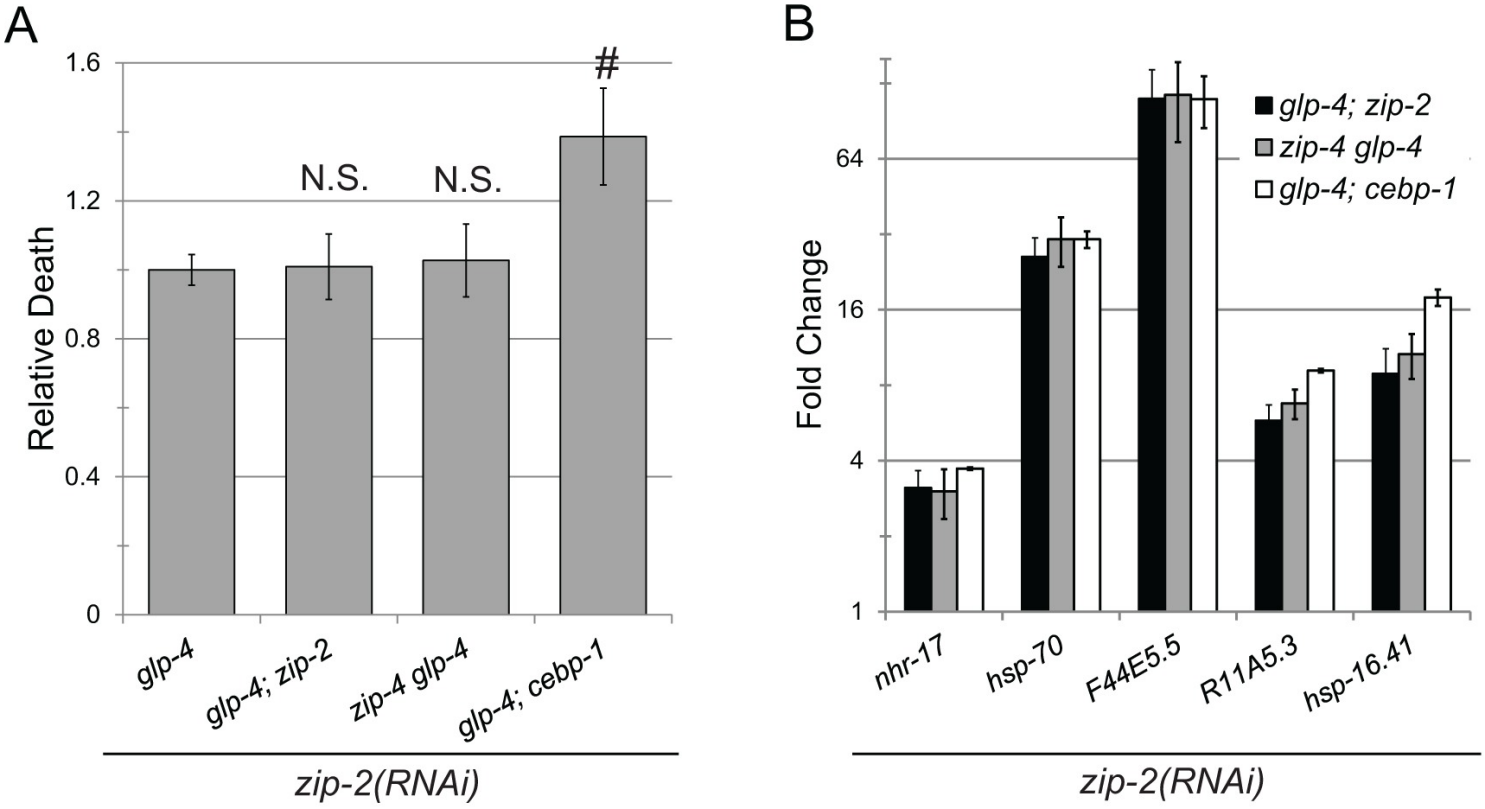
ZIP-2 and CEBP-1 have independent and overlapping innate immune functions. **(A)** Survival of *glp-4(bn2)*, *glp-4(bn2); zip-2(tm4248)*, *zip-4(tm1359) glp-4(bn2)*, and *glp-4(bn2); cebp-1(tm2807)* mutants grown on *zip-2(RNAi*) after exposure to *P*. *aeruginosa* in liquid. Statistical significance was determined via Student’s *t*-test, error bars represent SEM, hash represents *p*-value < 0.05, N.S. represents *p*>0.05. **(B)** Expression of ESRE genes in the same panel of worms as in **(A)** after exposure to Liquid Killing conditions for 12h. Fold changes were normalized to untreated genotypic cohorts.

The overlap between genes dependent upon ZIP-2 for their expression and upregulation by either phenanthroline or Liquid Killing, along with the remarkable sensitivity of ZIP-2 mutants to Liquid Killing [29], suggested that ZIP-2 may be involved in host defense against mitochondrial damage. Therefore, we hypothesized that ZIP-2 may be necessary for proper expression of ESRE genes. We tested this prediction by using qRT-PCR to assay ESRE gene activation in *zip-2(tm4248)* mutants after exposure to phenanthroline. Upregulation of all genes tested was attenuated (Fig 6A). However, neither an intragenic deletion (*tm4248*) nor *zip-2* RNAi in a *glp-4(bn2); zip-2(tm4248)* background were sufficient to completely abolish expression of ESRE genes (Figs 6A and 7B). It should be noted that *zip-2(RNAi)* has been reported to be at least as effective as either of the deletion alleles (*tm4248* or *tm4067*) in disrupting ZIP-2 function [56]. However, it remains at least formally possible that even the combination of the deletion and the RNAi knockdown do not result in a null phenotype.

Another possibility is that ZIP-2 exhibits at least partial genetic redundancy. Therefore, we searched the genome of *C*. *elegans* for other bZIP proteins that were closely related to ZIP-2 and found three additional C/EBP subfamily members: *zip-4*, *cebp-1*, and *cebp-2*. Interestingly, CEBP-2 was recently reported to function with ZIP-2 to mediate host defense against Slow Killing [57]. RNAi targeting any of these four bZIP family members significantly compromised survival in Liquid Killing (Fig 6B). To confirm the RNAi phenotype, and eliminate the possibility of off-target RNAi effects, we generated *glp-4(bn2); zip-2(tm4248)*, *zip-4(tm1359) glp-4(bn2)*, and *glp-4(bn2)*; *cebp-1(tm2807)* double mutants. *cebp-2* and *glp-4* are both located on LGI, complicating construction of the *cebp-2(tm5421) glp-4(bn2)* double mutant. Each of the three double mutants showed increased sensitivity to Liquid Killing and phenanthroline (Fig 6C and 6D). Loss of ZIP-2, ZIP-4, or CEBP-1 individually was sufficient to compromise ESRE gene induction (Fig 6E), confirming their involvement in this process.

To further test the interrelationship of *zip-2*, *zip-4*, and *cebp-1*, we used RNAi to knock down *zip-2* in *glp-4(bn2)*, *glp-4(bn2); zip-2(tm4248)*, *zip-4(tm1359) glp-4(bn2)*, and *glp-4(bn2)*; *cebp-1(tm2807)* strains. We assayed survival in Liquid Killing (Fig 7A) and ESRE gene induction (Fig 7B) in these backgrounds. Adding *zip-2(RNAi)* into double mutant backgrounds only showed a negative additive effect on survival of *glp-4(bn2); cebp-1(tm2807)* double mutants. At the same time, there was no evidence of any additive or synergistic interaction between *zip-2* and *cebp-1* in regulating the expression of ESRE genes. This suggests that at least some CEBP-1 function(s) relevant for survival in Liquid Killing are not shared with ZIP-2 and are independent of ESRE regulation. In the same vein, all of ZIP-4’s functions in Liquid Killing are within the same pathway(s) (or even the same complex) as those of ZIP-2.

Finally, we assayed *glp-4(bn2)*, *glp-4(bn2); zip-2(tm4248)*, *zip-4(tm1359) glp-4(bn2)*, and *glp-4(bn2)*; *cebp-1(tm2807)* mutants for the expression of a panel of genes that were upregulated under Slow Killing conditions (i.e., infections with *P*. *aeruginosa* on agar). As previously noted, ZIP-2 is critical for defense under these conditions [56]. While *zip-4* mutation failed to affect the expression of any of the genes tested, *cebp-1* mutation disrupted expression of one-third of the genes (the same number showed attenuation after *zip-2* mutation, S7A Fig). We further tested whether this gene family plays a role in defense by performing classical Slow Killing assays with mutant animals (S7B Fig). Mutation of either *zip-2* or *cebp-1* caused substantial decreases in survival, demonstrating a previously unappreciated role for CEBP-1 in promoting innate immune function.

### Regulation of ESRE genes by bZIP transcription factors is not a consequence of *glp-4(bn2)* mutation

In order to confirm that the phenotypes observed are not a consequence of the *glp-4*(*bn2*) allele, we assayed expression of the same panel of 8 ESRE genes in wild-type, *zip-4(tm1359)*, *zip-2(tm4248)*, and *cebp-1(tm2807)* worms after exposure to *P*. *aeruginosa* (Table 2, and see S10 Table for detailed data analysis). Consistent with our prior observations, ESRE genes were upregulated under Liquid Killing conditions. Upregulation was attenuated in each of the bZIP mutants tested.

**Table 2:** ESRE genes’ expression in wild-type background.

Gene	Name	N2/LK	*zip-2*/LK	*zip-4*/LK	*cebp-1*/LK
C02B4.2	*nhr-17*	40.6	10.2	N.S.	N.S.
C12C8.1	*hsp-70*	118.7	19.3	N.S.	N.S.
F44E5.5	F44E5.5	733.4	207.8	127.1	35.8
R11A5.3	R11A5.3	85.9	N.S.	N.S.	N.S.
Y46H3A.2	*hsp-16*.*41*	378.2	115.9	71.1	N.S.
C06B3.7	C06B3.7	Below Detection Threshold in All Conditions			
T27F6.8	T27F6.8	Below Detection Threshold in All Conditions			
F26A3.4	F26A3.4	4.5	2.8	N.S.	N.S.

LK–Liquid Killing;

N.S.–did not pass significance criteria

Next, we assayed survival of single, double, and triple bZIP mutants during Liquid Killing. Each genotype tested showed significant susceptibility to pathogen when compared to wild-type worms (S8A Fig), but only one double mutant (*cebp-2(tm5421)*; *zip-2 (tm4248))* was significantly more sensitive to the pathogen than the individual mutants comprising it (S8A Fig, marked with grey arrow). However, this double mutant strain appeared sickly (smaller size and with reduced fecundity) (S8B Fig). All other mutants were indistinguishable from wild-type worms when fed *E*. *coli*. These observations make it impossible to unambiguously claim that the increase in death under Liquid Killing conditions is specifically due to increases in sensitivity to *P*. *aeruginosa*.

## Discussion

The opportunistic, multi-host pathogen *P*. *aeruginosa* exhibits a variety of virulence mechanisms, and has been shown to cause at least five different types of killing in *C*. *elegans* [36]. Amongst these is Liquid Killing, a lethal, pyoverdine-mediated intoxication characterized by mitochondrial damage and a hypoxic response [24,29]. Since pyoverdine is also an essential component of mammalian infections [26–28,58], we assayed the response to *C*. *elegans* Liquid Killing to gain understanding about how hosts defend themselves against this type of insult.

Our data clearly demonstrate that the responses of *C*. *elegans* to pyoverdine intoxication or phenanthroline exposure are very different from genes activated by classical intestinal colonization (Slow Killing). Although there was a statistically significant overlap in transcriptional responses between Slow and Liquid Killing, the biological significance of this overlap is likely very low. For example, only eight genes were differentially regulated under both conditions and the Gene Ontology profiles are quite disparate. Perhaps most meaningfully, the PMK-1/p38 MAPK pathway, which is most responsible for resistance to intestinal colonization compromises the host's ability to survive pyoverdine intoxication [29].

In contrast, the host response to Liquid Killing was more similar to conventional stress responses, such as those induced by hypoxia or by heat shock. Much of the response to Liquid Killing, can be mimicked by phenanthroline, which has the same molecular function, but is structurally unrelated (Fig 3B). Combined, these data indicate that the host's defensive mechanisms are focused on mitigating the damage caused by iron removal that is catalyzed by these different compounds. This demonstrates a particularly nuanced response to *P*. *aeruginosa* exposure, wherein the host defense identifies and attempts to ameliorate the most important virulence determinants.

Analysis of the promoter regions of genes upregulated specifically in response to Liquid Killing revealed a previously-identified, 11-nucleotide motif referred to as ESRE [40]. This motif has an interesting history, as it has seemingly been independently discovered at least seven times, and is responsive to hypoxia, ethanol, heat, and oxidative stress [40,42,53,59–62]. Intriguingly, all nine ESRE-activating conditions identified in this and other papers (pyoverdine, phenanthroline, rotenone, antimycin A, ethanol, hypoxia, heat shock, oxidative stress, and *spg-7* (*RNAi*)) disrupt mitochondrial function. In contrast, stressors with other modalities like hygromycin B, tunicamycin, bortezomib, or exotoxin A did not result in ESRE genes’ activation. The identity of the DAMP(s) (e.g., substrates or products of oxidative phosphorylation, defective protein import or overproduction of reactive oxygen species) remains to be elucidated.

A recent report observed a strong similarity between the ESRE motif and the consensus binding site for the EOR-1 transcription factor, and speculated that EOR-1 may play a previously unknown role in longevity after mitochondrial disruption [61]. There are several compelling reasons to doubt that EOR-1 is required for upregulation of ESRE genes, however. First, although 8 of the 11 nucleotides in ESRE match the EOR-1 consensus, the 3 nucleotides in the center showed much higher conservation in ESRE than are observed in the EOR-1 consensus, suggesting the presence of an additional specificity determinant for ESRE binding. Second, small HSPs, such as the HSP-16 family, are enriched in ESRE sites and are amongst the most strongly upregulated genes after Liquid Killing. EOR-1 and EOR-2 have clearly been shown to repress, rather than induce, the expression of this gene family [63]. Third, expression of ESRE-containing genes was indistinguishable between strains with or without mutations in *eor-1* or *eor-2* (S9 Fig). Combined, these observations make it unlikely that EOR-1 is the factor driving expression of ESRE genes after mitochondrial insult.

In contrast, the bZIP family transcription factors ZIP-2, ZIP-4, and CEBP-1 do seem to regulate the expression of ESRE genes, as mutation of any one of them significantly diminishes the upregulation of ESRE genes after exposure to iron chelating compounds (Fig 6E). These three transcription factors also confer resistance to both Liquid Killing and phenanthroline (Fig 6C and 6D). It is worth noting that bZIP family members have distinct individual roles. For example, ZIP-2 and CEBP-1 are both required for survival in Liquid Killing and play additional, unique roles independent of ESRE regulation. This is evidenced by the enhanced sensitivity to *P*. *aeruginosa* in Liquid Killing observed for a *glp-4(bn2); zip-2(RNAi); cebp-1(tm2807)* triple mutant. ZIP-2 and CEBP-1, but not ZIP-4, are also required for survival during infection with *P*. *aeruginosa* on agar plates. This is, to our knowledge, the first report suggesting that CEBP-1 promotes host defense against *P*. *aeruginosa* infection in Slow Killing. Studies using single, double, and triple mutants in a wild-type background eliminated the possibility that the phenomena we observed were related to the absence of the germline (caused by the *glp-4(bn2)* lesion). Further study of the ESRE network, its mechanisms, and this bZIP protein family are currently underway in our lab.

Finally, our data support the existence of a phylogenetically conserved mitochondrial surveillance network that promotes organismal survival in the context of innate immune and stress response functions. In contrast to ER surveillance, where a wealth of knowledge has been discovered (including pathways monitoring protein folding or glycosylation [64], etc.), mitochondrial surveillance is relatively new, especially in mammals. Due to its diverse functions, its extracellular (i.e., non-self) origins, and its roles in many cell death pathways, mitochondrial surveillance is almost certainly under intricate and detailed control. This research has been spearheaded in *C*. *elegans*, where important discoveries about surveillance of the mitochondrial unfolded protein response [46,65–68] have been made. In many cases, activation of these pathways results in transcription of pathogen defense pathways [48,66], a process called cellular-Surveillance-Activated Detoxification and Defenses (cSADD) [48].

cSADD has been hypothesized to help identify otherwise covert microbial activities compromising host health. We favor an alternative hypothesis. The cSADD pathways and the ESRE network may have originally arisen to monitor intracellular processes and only later were integrated into the innate immune system. Although stress response and innate immunity are generally conceived of as separate phenomena (abiotic vs biotic), this dichotomy is a conceptual device, and may not accurately reflect events occurring at the cellular level. In practice, both stress response and innate immunity recognize the same perturbations: molecules that are out of their normal context (e.g., misfolded proteins, disruptions of mitochondrial integrity and function, dsRNA of an inappropriate length, etc.). This is evidenced by the extensive overlap of the cellular processes involved in both defense mechanisms. Broadening our understanding to include both processes under the same conceptual framework is likely to lead to new hypotheses and discoveries.

## Methods

### *C*. *elegans* strains

All *C*. *elegans* strains were maintained on nematode growth medium (NGM) seeded with *Escherichia coli* strain OP50 as a food source and were maintained at 15°C [69], unless otherwise noted. *C*. *elegans* strains used in this study included N2 Bristol (wild-type), SS104 [*glp-4*(*bn2*)] [70], CZ8920 [*cebp-1*(*tm2807*)], ERT061 [*zip-2*(*tm4248*)], ERT214 [*cebp-2*(*tm5421*)], ERT216 [*cebp-2*(*tm5421*); *zip-2*(*tm4248*)], NVK56 [*zip-4*(*tm1359*)], NVK77 [*zip-4*(*tm1359*); *zip-2*(*tm4248*)], NVK81 [*zip-2*(*tm4248*); *cebp-1*(*tm2807*)], NVK83 [*zip-4*(*tm1359*); *cebp-1*(*tm2807*)], NVK87 [*zip-4*(*tm1359*); *zip*-2(*tm4248*); *cebp-1*(*tm2807*)], NVK98 [*glp-4*(*bn2*); *houIs002* |*pJY323*(P*hsp-16*.*1*::GFP); *pRF4*|], NVK214 [*glp-4(bn2); zip-2*(*tm4248*)], NVK215 [*zip-4(tm1359*) *glp-4*(*bn2*)], NVK216 [*glp-4*(*bn2*); *cebp-1*(*tm2807*)], WY753 (*fdEx142* |*pJY323* [P_*hsp-16*.*1*_::*GFP*]; *pRF4* [*rol-6*(*gf*)]|) [43], WY756 (*fdEx139* |*pJY312* [P_*hsp-16*.*1(dd)*_::*GFP*]; pRF4|) [43].

Media conditions include NGM [69], standard nematode growth medium; SK, modified NGM used for plate-based infection with *P*. *aeruginosa* [71]; LK, modified liquid NGM used for Liquid Killing [71]. Prior to experiments, worms were synchronized by hypochlorite isolation of eggs from gravid adults, followed by hatching of eggs in S basal. L1 larvae were transferred to NGM plates seeded with OP50 or NGM plates supplemented with 25 μg/ml carbenicillin and 1mM IPTG that were seeded with appropriate RNAi strains. After transfer, worms were grown overnight at 15°C, and then shifted to 25°C for 44–48 hours prior to use. For strains that did not have a sterile background, worms were reared on *cdc-25*.*1(RNAi)* prior to use in assays to guarantee sterility. Young adult worms were used for all assays. RNAi treatment was performed by seeding 4500 synchronized L1 larvae onto 10 cm NGM plates supplemented with carbenicillin and 1 mM IPTG. Worms were used when they reached the young adult stage.

### Bacterial strains

*Pseudomonas aeruginosa* strain PA14 is a previously described clinical isolate [31]. PA14 pyoverdine mutants (*pvdF* and *pvdP*) were obtained from a transposon insertion library [72] and were verified by DNA sequencing. RNAi experiments in this study were performed by using RNAi-competent OP50(xu363) [73], except for RNAi of ESRE-containing effectors, which were performed using HT115(DE3)-based strains. RNAi clones were obtained by purifying appropriate plasmids from the Ahringer RNAi library [44,45] and were sequenced prior to use.

### *C*. *elegans* pathogenesis and chemical exposure assays

Slow and Liquid Killing assays were performed essentially as previously described, except that synchronized young adult worms were used for Slow Killing [71]. To determine phenanthroline sensitivity, the Liquid Killing assay was modified as follows: first, *P*. *aeruginosa* PA14 was substituted with *E*. *coli* OP50 at a final density of OD_600_ = 0.03. Second, 1,10-phenanthroline was added at a final concentration of 1 mM.

Prior to exposure to small molecules, synchronized young adult worms were washed from NGM plates seeded with OP50, and resuspended in S basal supplemented with 50 μM antimycin A (Sigma), 50 μM rotenone (Sigma), 80 μg/mL hygromycin B, 60 μM tunicamycin (TOCRIS Bioscience), or 12.5 μM bortezomib (LC Laboratories) in the presence of OP50. Worms were exposed for 8 hours prior to RNA harvest (see below).

The ToxA sensitivity assay was performed as previously described, except that worms were reared on the toxin-expressing bacteria for 8 hours. *E*. *coli* strain expressing empty pET100 vector and pET100 expressing ToxA were grown, induced, and seeded on NGM plates containing carbenicillin and IPTG as described [49]. Synchronized young adult worms were washed from NGM plates seeded with OP50 and were rinsed three times to remove residual bacteria. Approximately 5000 worms were added to each lawn and were incubated for 8 hours at 25°C and then RNA was harvested (see below).

At least three biological replicates (defined as three independent trials) were performed for each experiment. *p*-values were determined via Student’s *t*-test.

### Microarray and motif discovery

Microarray analysis and motif discovery were performed essentially as previously described [74]. Briefly, RNA was isolated from worms after 8 h exposure to: *E*. *coli* strain OP50 on NGM media plates, *E*. *coli* OP50 on SK media plates, *E*. *coli* OP50 in LK media, infected with *P*. *aeruginosa* strain PA14 on SK media plates, exposed to *P*. *aeruginosa* PA14 in liquid, treated with vehicle (1% v/v DMSO) in LK media, or treated with 1 mM 1,10-phenanthroline in LK media. RNA was harvested via phenol-chloroform extraction and cleaned up with an RNEasy kit (Qiagen). Triplicate samples were hybridized to Affymetrix *C*. *elegans* genome arrays. Gene expression levels were determined via GCRMA in Bioconductor. Genes were considered differentially expressed on the basis of a modified Wilcoxon rank test, as previously described (i.e., fold change > 2, modified Wilcoxon rank > 1.5) [74].

For conditions reported elsewhere (Table 1, S3 Fig), differentially regulated genes were defined by the original publication. *p*-values for Venn diagrams were calculated using hypergeometric probability. *p*-values for overrepresentation of Gene Ontology categories were derived by DAVID v. 6.7 (*david*.*abcc*.*ncifcrf*.*gov/*), * denotes multiple hypothesis correction using Benjamini-Hochberg method. Microarray data has been deposited in the GEO database with the access number GSE55422. Access link: https://www.ncbi.nlm.nih.gov/geo/query/acc.cgi?token=wtgzsmcojjwdjmr&acc=GSE55422

### Quantitative Reverse Transcriptase PCR (qPCR)

RNA purification and qPCR were performed as previously described [24]. For human ESRE genes, targets were identified using a reciprocal BLAST search as previously described [42]. Prior to RNA extraction, human cells were treated with 1,10-phenanthroline, ciclopirox olamine, or DMSO. Primer sequences are available upon request. For each experiment at least three biological replicates (defined as three independent trials) were used. *p*-values were derived from Student’s *t*-test.

### Imaging

Fluorescence and bright-field micrographs of WY753 and WY756 reporter strains [43] were captured from slide-mounted, young adults that had previously been exposed to 1% DMSO (v/v) or 1 mM 1,10-phenanthroline in 1% DMSO (v/v) for 12 hours in S basal in the presence of *E*. *coli* OP50. Experiments were performed at 20°C to minimize any fluorescence resulting from heat shock. Images were acquired and fluorescence was quantified using a Zeiss AXIO Imager ZI microscope with a Zeiss AxioCam HRm camera and Axiovision 4.6 (Zeiss) software. Fluorescence was quantified from at least 50 worms per condition per biological replicate. At least three biological replicates (defined as three independent trials) were performed. *p*-values were determined by Student’s *t*-test.

For visualization of NVK98 young adults exposed to *P*. *aeruginosa* or *E*. *coli* in 96-well plates, a Cytation 5 Cell Imaging Multi-Mode Reader (BioTek Instruments) was used. All imaging was performed with identical settings.

## Supporting information

S1 Fig Validation of Liquid Killing microarray expression data. Expression levels of 11 upregulated genes as determined by microarray (*x*-axis) and qPCR (*y*-axis). The line of best-fit and its correlation coefficient are shown. Fold changes were normalized to untreated genotypic cohorts.
(PDF)

S2 Fig Presence of the *glp-4(bn2)* allele does not substantially alter the host defense response. **(A,C)** Expression levels of 12 genes upregulated in Liquid Killing in either wild-type (N2) background or in the presence of the sterility-inducing *glp-4(bn2)* allele after exposure to *P*. *aeruginosa* in liquid **(A)** or on agar plates **(C)**. **(B,D)** Expression levels of 7 genes upregulated in Slow Killing in either wild-type (N2) background or in the presence of the sterility-inducing *glp-4(bn2)* allele after exposure to *P*. *aeruginosa* in liquid **(B)** or on agar plates **(D)**. Fold changes were normalized to untreated genotypic cohorts. Error bars represent SEM.
(PDF)

S3 Fig Clustering of genes upregulated in pyoverdine-dependent Liquid Killing. **(A)** Expression patterns of genes were used for hierarchical clustering across eleven different microarray conditions. Host and microbial strains, media conditions (see Methods for details), and references are shown. **(B)** A cluster showing enrichment of genes upregulated specifically in Liquid Killing is shown. Conditions (*x*-axis) correspond to the eleven data sets listed in **(A)**. The heavy red line indicates mean expression level of genes within the cluster.
(PDF)

S4 Fig A subset of ESRE genes is necessary for survival during Liquid Killing. A panel of ESRE-containing genes were disrupted via RNAi and worms were subsequently exposed to *P*. *aeruginosa* (Liquid Killing). Asterisks represent RNAi knockdowns with significantly higher death rate (*p* < 0.05) in 4/4 replicates. Hashes represent RNAi knockdowns with significantly higher death rate (*p* < 0.05) in 3/4 replicates. Average survival is plotted for all biological replicates. Error bars represent standard deviation.
(PDF)

S5 Fig The ESRE reporter gene is activated by *P*. *aeruginosa*. NVK98 young adult worms expressing *p*_*hsp-16*_::GFP were exposed to either *E*. *coli* OP50 or *P*. *aeruginosa* PA14 under Liquid Killing conditions for 24 h **(A)** or 48 h **(B)** in 96-well plates. Images were taken under identical settings. Scale bars represent 1000 μm.
(PDF)

S6 Fig ESRE gene activation is specific. Expression of ESRE-containing and non-ESRE containing genes (red label) after treatment with a proteasomal inhibitor (bortezomib), an activator of the ER unfolded protein response (tunicamycin), or a translational inhibitor (*E*. *coli* expressing Exotoxin A from *P*. *aeruginosa*). Fold changes are normalized to the solvent control.
(PDF)

S7 Fig bZIP family members show different roles in innate immunity. **(A)** Expression of a panel of genes upregulated during *P*. *aeruginosa* infection on agar (i.e., Slow Killing) in *glp-4(bn2)*, *glp-4(bn2); zip-2(tm4248)*, *zip-4(tm1359) glp-4(bn2)*, and *glp-4(bn2); cebp-1(tm2807)* mutants. Expression values were normalized to untreated genotypic cohorts. Error bars represent SEM, asterisks show *p*-value < 0.01, hashes show *p*-value < 0.05. **(B)** Slow kill assays of *glp-4(bn2)*, *glp-4(bn2); zip-2(tm4248)*, *zip-4(tm1359) glp-4(bn2)*, and *glp-4(bn2); cebp-1(tm2807)* mutants. A representative replicate (one of three) is shown.
(PDF)

S8 Fig bZIP family members are sensitive to Liquid Killing. **(A)** Survival of a panel of bZIP mutants exposed to *P*. *aeruginosa* (Liquid Killing). **(B)** Representative images of bZIP mutants on a non-pathogenic food source (*E*. *coli* OP50). Statistical significance was determined via Student’s *t*-test, error bars represent SEM, asterisks represent *p*-value < 0.01. The grey arrow indicates the *cebp-2; zip-2* double mutant, which shows significantly more death than either single mutant.
(PDF)

S9 Fig EOR-1 and EOR-2 are dispensable for ESRE gene expression. Expression of a panel of ESRE genes in *glp-4(bn2)*, *glp-4(bn2); eor-1(cs28)*, and *glp-4(bn2); eor-2(cs42)*. Expression values were normalized to untreated genotypic cohorts. Error bars represent SEM.
(PDF)

S1 Table List of genes upregulated during Liquid or Slow Killing. (XLSX)

S2 Table Genes upregulated by both Liquid and Slow Killing. (DOCX)

S3 Table ESRE motif is specifically enriched in Liquid Killing. (DOCX)

S4 Table List of genes upregulated during phenanthroline exposure. (XLSX)

S5 Table GO categories enriched in genes shared by Liquid Killing and phenanthroline. (DOCX)

S6 Table GO categories for 59 genes specific to Liquid Killing. (DOCX)

S7 Table GO categories for 509 phenanthroline-specific genes. (DOCX)

S8 Table Host response to Liquid Killing is similar to treatment with phenanthroline. (DOCX)

S9 Table List of selected human genes with ESRE sites. (DOCX)

S10 Table ESRE genes panel expression in wild-type and bZip mutants. (XLSX)
