# Supplementary figures and images for: A conserved mitochondrial surveillance pathway is required for defense against Pseudomonas aeruginosa
Source: PLoS Genet. 2017 Jun 29;13(6):e1006876. doi: 10.1371/journal.pgen.1006876 (PMC5510899; doi:10.1371/journal.pgen.1006876)

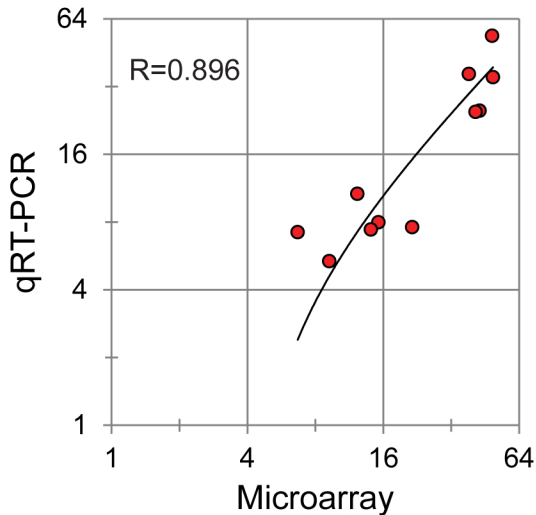

S1 Fig. Validation of Liquid Killing microarray expression data

Supplement: S1 Fig — Expression levels of 11 upregulated genes as determined by microarray (x-axis) and qPCR (y-axis). The line of best-fit and its correlation coefficient are shown. Fold changes were normalized to untreated genotypic cohorts. (PDF) [file pgen.1006876.s001.pdf]

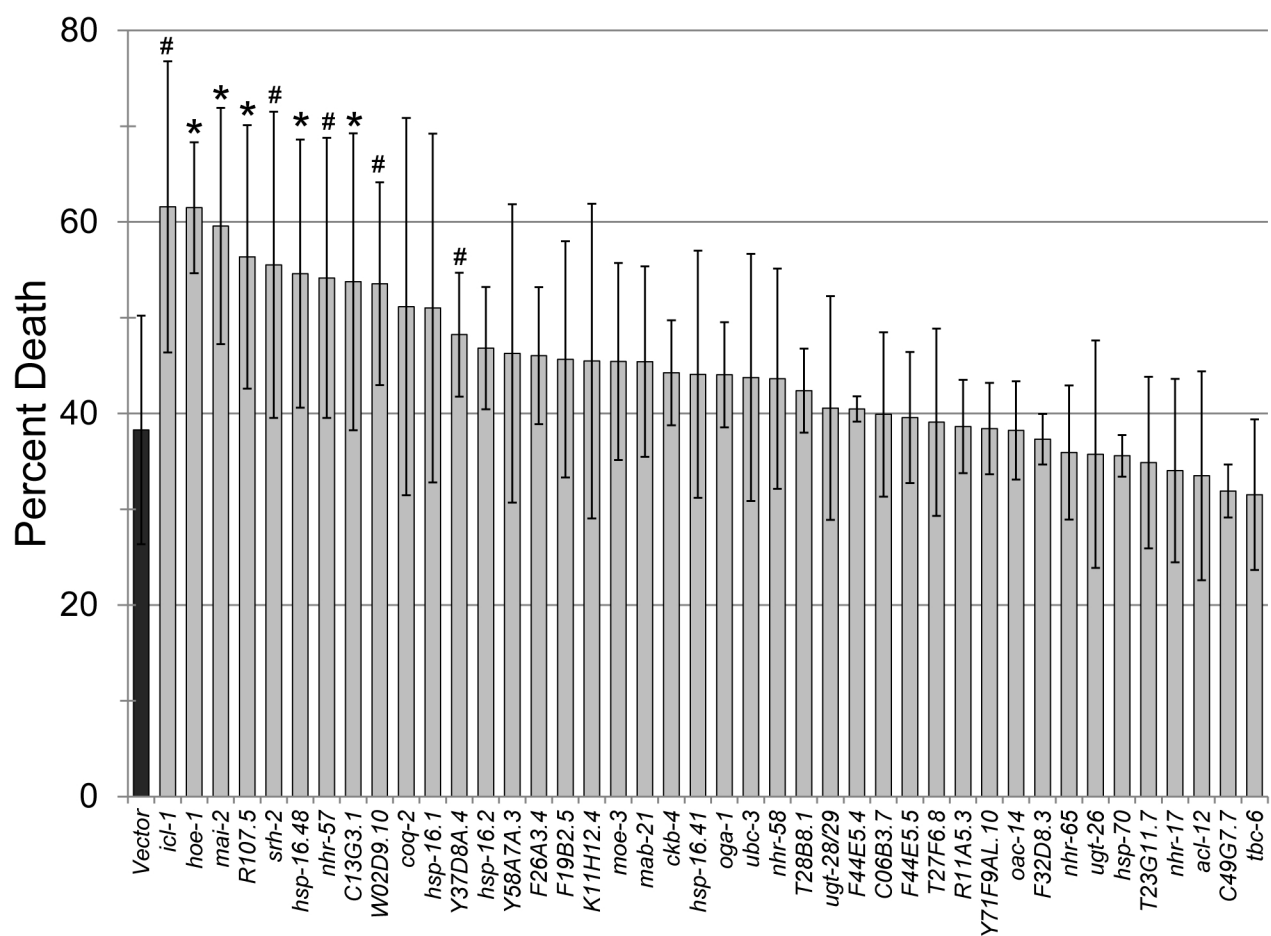

S4 Fig. A subset of ESRE genes is necessary for survival during Liquid Killing

Supplement: S4 Fig — A panel of ESRE-containing genes were disrupted via RNAi and worms were subsequently exposed to P. aeruginosa (Liquid Killing). Asterisks represent RNAi knockdowns with significantly higher death rate (p < 0.05) in 4/4 replicates. Hashes represent RNAi knockdowns with significantly higher death rate (p < 0.05) in 3/4 replicates. Average survival is plotted for all biological replicates. Error bars represent standard deviation. (PDF) [file pgen.1006876.s004.pdf]

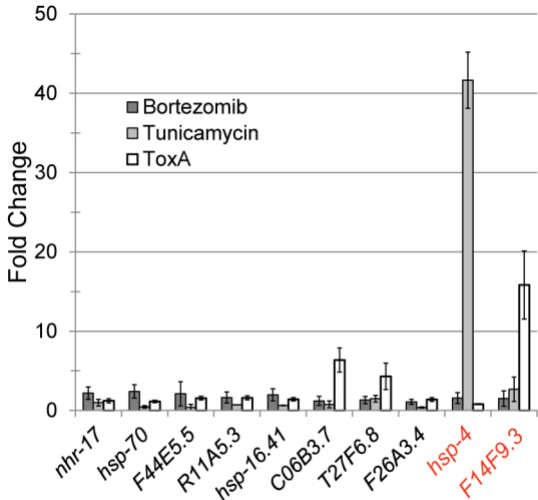

S6 Fig. ESRE gene activation is specific

Supplement: S6 Fig — Expression of ESRE-containing and non-ESRE containing genes (red label) after treatment with a proteasomal inhibitor (bortezomib), an activator of the ER unfolded protein response (tunicamycin), or a translational inhibitor (E. coli expressing Exotoxin A from P. aeruginosa). Fold changes are normalized to the solvent control. (PDF) [file pgen.1006876.s006.pdf]

A

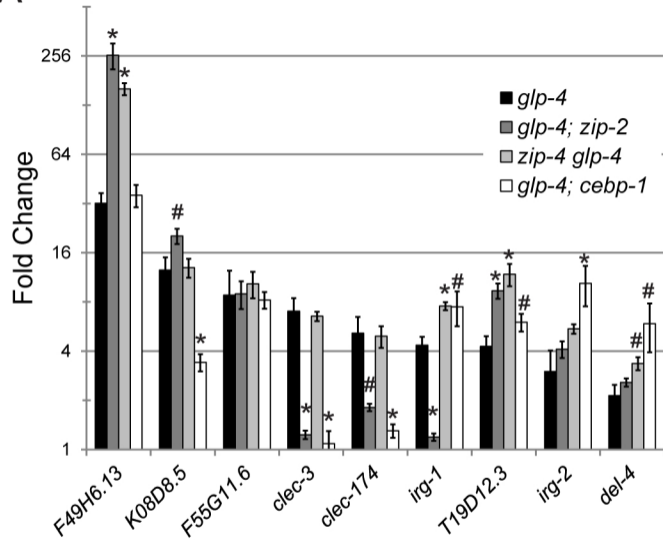

B

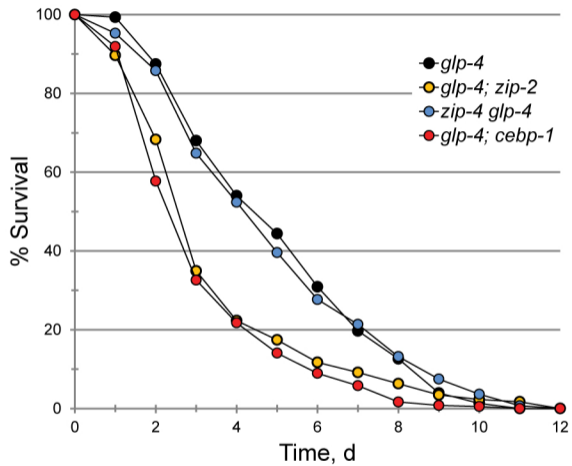

S7 Fig. bZIP family members show different roles in innate immunity

Supplement: S7 Fig — (A) Expression of a panel of genes upregulated during P. aeruginosa infection on agar (i.e., Slow Killing) in glp-4(bn2), glp-4(bn2); zip-2(tm4248), zip-4(tm1359) glp-4(bn2), and glp-4(bn2); cebp-1(tm2807) mutants. Expression values were normalized to untreated genotypic cohorts. Error bars represent SEM, asterisks show p-value < 0.01, hashes show p-value < 0.05. (B) Slow kill assays of glp-4(bn2), glp-4(bn2); zip-2(tm4248), zip-4(tm1359) glp-4(bn2), and glp-4(bn2); cebp-1(tm2807) mutants. A representative replicate (one of three) is shown. (PDF) [file pgen.1006876.s007.pdf]

A

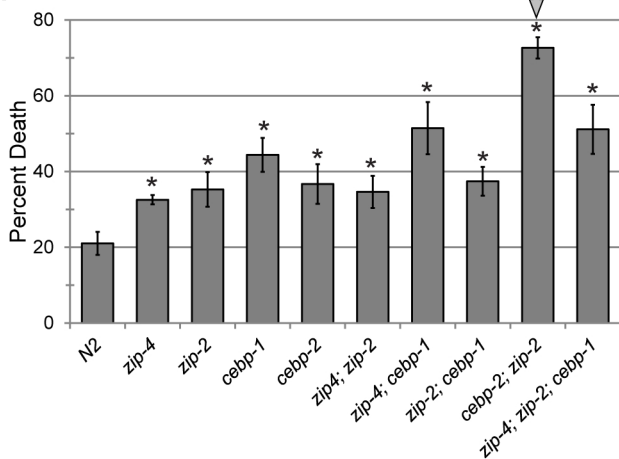

B

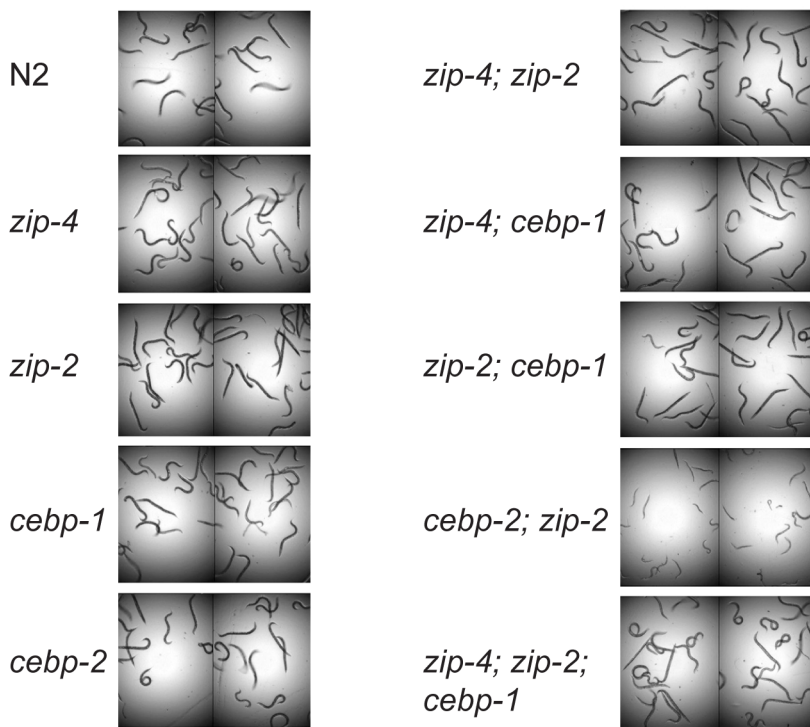

S8 Fig. bZIP family members are sensitive to Liquid Killing

Supplement: S8 Fig — (A) Survival of a panel of bZIP mutants exposed to P. aeruginosa (Liquid Killing). (B) Representative images of bZIP mutants on a non-pathogenic food source (E. coli OP50). Statistical significance was determined via Student’s t-test, error bars represent SEM, asterisks represent p-value < 0.01. The grey arrow indicates the cebp-2; zip-2 double mutant, which shows significantly more death than either single mutant. (PDF) [file pgen.1006876.s008.pdf]

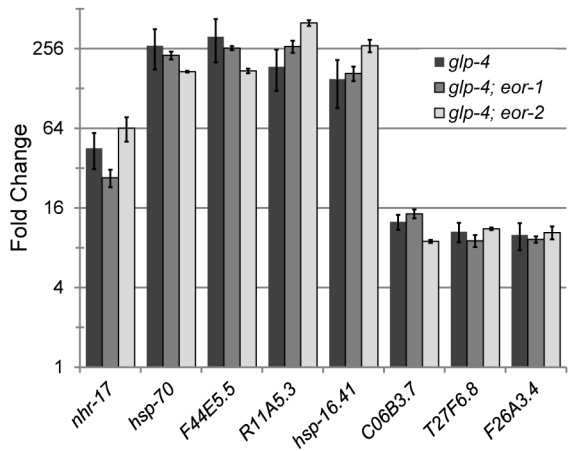

S9 Fig. EOR-1 and EOR-2 are dispensable for ESRE gene expression

Supplement: S9 Fig — Expression of a panel of ESRE genes in glp-4(bn2), glp-4(bn2); eor-1(cs28), and glp-4(bn2); eor-2(cs42). Expression values were normalized to untreated genotypic cohorts. Error bars represent SEM. (PDF) [file pgen.1006876.s009.pdf]
