# Supplementary material for: A conserved mitochondrial surveillance pathway is required for defense against Pseudomonas aeruginosa
Source: PLoS Genet. 2017 Jun 29;13(6):e1006876. doi: 10.1371/journal.pgen.1006876 (PMC5510899; doi:10.1371/journal.pgen.1006876)
Supplement: S2 Fig — (A,C) Expression levels of 12 genes upregulated in Liquid Killing in either wild-type (N2) background or in the presence of the sterility-inducing glp-4(bn2) allele after exposure to P. aeruginosa in liquid (A) or on agar plates (C). (B,D) Expression levels of 7 genes upregulated in Slow Killing in either wild-type (N2) background or in the presence of the sterility-inducing glp-4(bn2) allele after exposure to P. aeruginosa in liquid (B) or on agar plates (D). Fold changes were normalized to untreated genotypic cohorts. Error bars represent SEM. (PDF) [file pgen.1006876.s002.pdf]

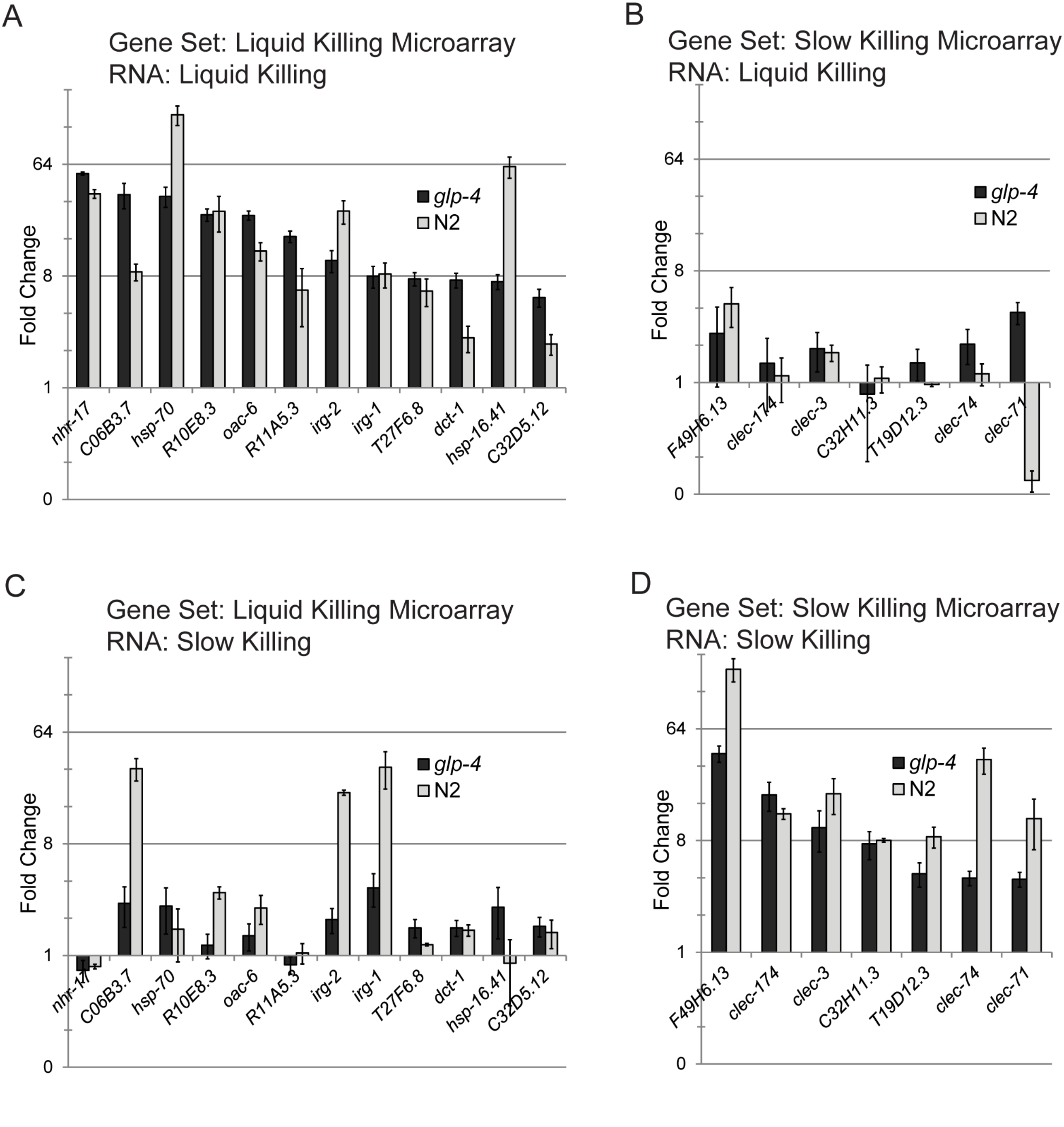

S2 Fig. Presence of the *glp-4(bn2)* allele does not substantially alter the host defense response
