# Supplementary material for: A conserved mitochondrial surveillance pathway is required for defense against Pseudomonas aeruginosa
Source: PLoS Genet. 2017 Jun 29;13(6):e1006876. doi: 10.1371/journal.pgen.1006876 (PMC5510899; doi:10.1371/journal.pgen.1006876)
Supplement: S3 Fig — (A) Expression patterns of genes were used for hierarchical clustering across eleven different microarray conditions. Host and microbial strains, media conditions (see Methods for details), and references are shown. (B) A cluster showing enrichment of genes upregulated specifically in Liquid Killing is shown. Conditions (x-axis) correspond to the eleven data sets listed in (A). The heavy red line indicates mean expression level of genes within the cluster. (PDF) [file pgen.1006876.s003.pdf]

A

Condition	Worm strain	Bacterial strain	Description	PMID
1	*glp-4(bn2)*	*P. aeruginosa* PA14	LK, Liquid	This study
2	*glp-4(bn2)*	*E. coli* OP50	LK, Liquid	This study
3	*glp-4(bn2)*	*E. coli* OP50	NGM, Plate	This study
4	*glp-4(bn2)*	*E. coli* OP50	SK, Plate	This study
5	*glp-4(bn2)*	*P. aeruginosa* PA14	SK, Plate	This study
6	N2	*E. coli* OP50	BHI, Plate	21731485
7	N2	*C. albicans* DAY185	BHI, Plate	21731485
8	*fer-15(b26ts);fem-1(hc17)*	*E. coli* OP50	TSA, plate	20617181
9	*fer-15(b26ts);fem-1(hc17)*	*S. aureus* RN6390	TSA, plate	20617181
10	N2	*E. coli* OP50	SK, Plate	20133945
11	N2	*Y. pestis* KIM5	SK, Plate	20133945

B

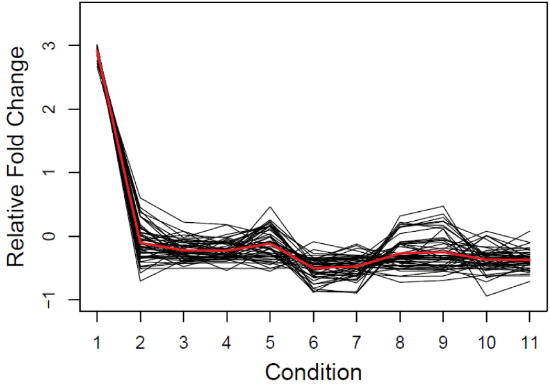

S3 Fig. Clustering of genes upregulated in pyoverdine-dependent liquid killing
