# Supplementary material for: A conserved mitochondrial surveillance pathway is required for defense against Pseudomonas aeruginosa
Source: PLoS Genet. 2017 Jun 29;13(6):e1006876. doi: 10.1371/journal.pgen.1006876 (PMC5510899; doi:10.1371/journal.pgen.1006876)
Supplement: S5 Fig — NVK98 young adult worms expressing phsp-16::GFP were exposed to either E. coli OP50 or P. aeruginosa PA14 under Liquid Killing conditions for 24 h (A) or 48 h (B) in 96-well plates. Images were taken under identical settings. Scale bars represent 1000 μm. (PDF) [file pgen.1006876.s005.pdf]

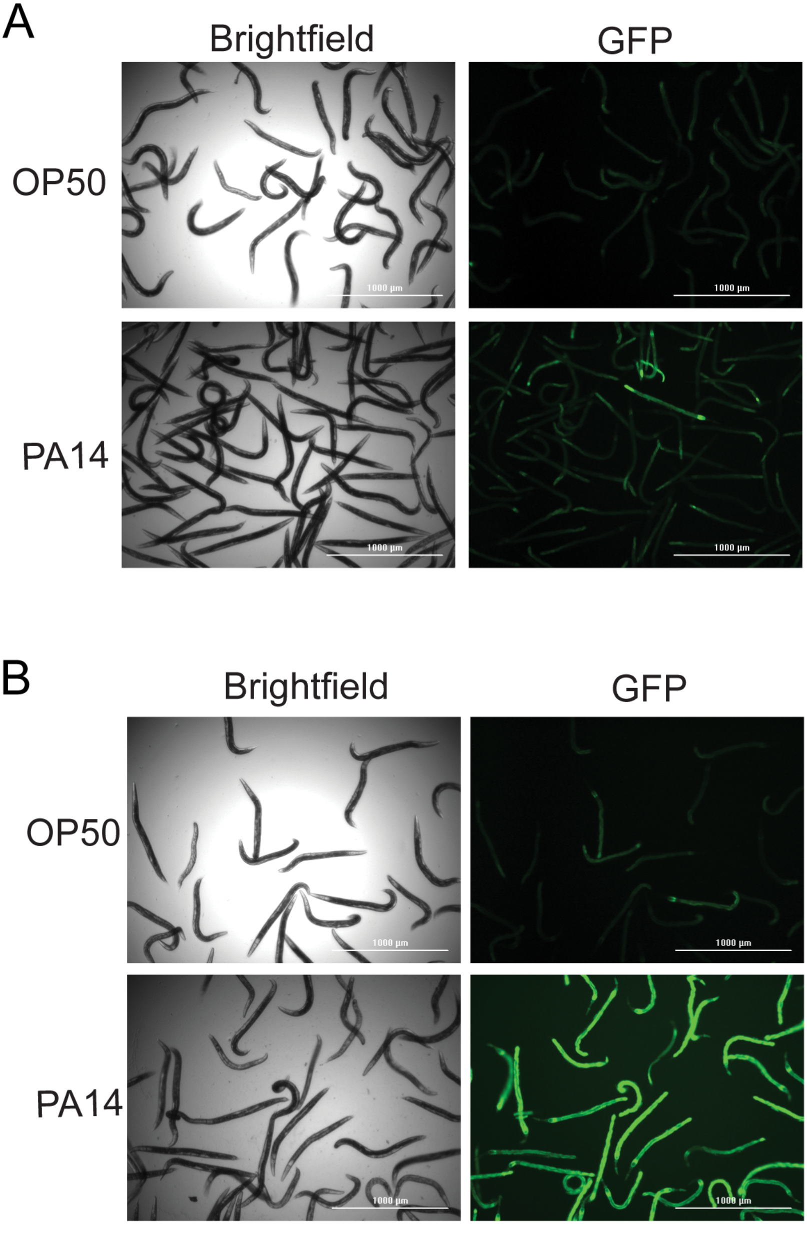

S5 Fig. The ESRE reporter gene is activated by *P. aeruginosa*
