# Supplementary material for: A conserved mitochondrial surveillance pathway is required for defense against Pseudomonas aeruginosa
Source: PLoS Genet. 2017 Jun 29;13(6):e1006876. doi: 10.1371/journal.pgen.1006876 (PMC5510899; doi:10.1371/journal.pgen.1006876)
Supplement: S2 Table — (DOCX) [file pgen.1006876.s011.docx]

**Table S2. Genes Upregulated by both Liquid and Slow Killing**

|  |  |  |  |  |  |
| --- | --- | --- | --- | --- | --- |
| **Affy ID** | **Gene** | **Name** | **Fold LK** | **Fold SK** | **Description** |
| 182733_at | C31A11.5 | *oac-6* | 41.06 | 2.09 | O-ACyltransferase homolog |
| 179226_at | C06B3.7 |  | 38.38 | 2.65 | An ortholog of human EPHX1 (epoxide hydrolase 1) |
| 183876_at | C07G3.2 | *irg-1* | 14.74 | 6.18 | Infection-response gene. May be ZIP-2 or PMK-1 dependent |
| 192229_s_at | Y38C9A.2 | *cgp-1* | 7.45 | 2.66 | An ortholog of human GTPBP1 (GTP binding protein 1) |
| 182815_at | T24C4.4 |  | 5.80 | 9.70 | Innate immune effector |
| 176295_at | Y22D7AR.9 | *fbxa-74* | 5.59 | 5.91 | F-box A protein |
| 176135_s_at | Y119D3B.18 | *fbxa-91* | 4.91 | 2.32 | F-box A protein, possibly involved in protein-protein interactions |
| 179854_at | Y47D3A.2 | *fbxa-128* | 3.04 | 4.41 | F-box A protein |

LK – Liquid Killing; SK – Slow Killing
