# Supplementary material for: A conserved mitochondrial surveillance pathway is required for defense against Pseudomonas aeruginosa
Source: PLoS Genet. 2017 Jun 29;13(6):e1006876. doi: 10.1371/journal.pgen.1006876 (PMC5510899; doi:10.1371/journal.pgen.1006876)
Supplement: S3 Table — (DOCX) [file pgen.1006876.s012.docx]

**Table S3. ESRE Motif is Specifically Enriched in Liquid Killing**

| **Category** | **% Genes**  **with Motif** | ***p*-value** |
| --- | --- | --- |
| *P. aeruginosa* -Liquid, Cluster | **43.9** | 1.1X10^-10^ |
| *P. aeruginosa*-Liquid | **28.0** | 1.9X10^-14^ |
| *E. coli*-Liquid | 8.1 | 0.503 |
| *P. aeruginosa*-Agar | 2.5 | 0.999 |
| *S.aureus* | 5.8 | 0.907 |
| *C.albicans* | 9.7 | 0.303 |
| Genome (background) | 8.0 |  |
