# Supplementary material for: A conserved mitochondrial surveillance pathway is required for defense against Pseudomonas aeruginosa
Source: PLoS Genet. 2017 Jun 29;13(6):e1006876. doi: 10.1371/journal.pgen.1006876 (PMC5510899; doi:10.1371/journal.pgen.1006876)
Supplement: S5 Table — (DOCX) [file pgen.1006876.s014.docx]

**Table S5. GO Categories Enriched in Genes Shared by Liquid Killing and Phenanthroline**

| **Description** | **Count** | **Enrichment** | ***p*-value*** |
| --- | --- | --- | --- |
| Heat shock protein 70 | 4 | 68.1 | 0.0037 |
| Alpha crystallin/Hsp20 domain | 4 | 37.8 | 0.0060 |
| Stress response | 6 | 13.0 | 0.0038 |
| Endoplasmic reticulum | 5 | 9.5 | 0.0299 |
| Oxidoreductase | 8 | 5.0 | 0.0203 |
| Zinc | 13 | 3.3 | 0.0147 |
| Metal-binding | 19 | 2.9 | 0.0051 |

**p*-value was calculated using Benjamini-Hochberg correction
