# Supplementary material for: A conserved mitochondrial surveillance pathway is required for defense against Pseudomonas aeruginosa
Source: PLoS Genet. 2017 Jun 29;13(6):e1006876. doi: 10.1371/journal.pgen.1006876 (PMC5510899; doi:10.1371/journal.pgen.1006876)
Supplement: S6 Table — (DOCX) [file pgen.1006876.s015.docx]

**Table S6. GO Categories for 59 Genes Specific to Liquid Killing**

| **Description** | **Count** | **Enrichment** | ***p*-value*** |
| --- | --- | --- | --- |
| Transferase | 12 | 4.47 | 0.002083 |

**p*-value was calculated using Benjamini-Hochberg correction
