# Supplementary material for: A conserved mitochondrial surveillance pathway is required for defense against Pseudomonas aeruginosa
Source: PLoS Genet. 2017 Jun 29;13(6):e1006876. doi: 10.1371/journal.pgen.1006876 (PMC5510899; doi:10.1371/journal.pgen.1006876)
Supplement: S7 Table — (DOCX) [file pgen.1006876.s016.docx]

**Table S7. GO Categories for 509 Phenanthroline-Specific Genes**

| **Description** | **Count** | **Enrichment** | ***p*-value*** |
| --- | --- | --- | --- |
| Fungicide | 3 | 30.7 | 3.7E-02 |
| NADH:flavin oxidoreductase/NADH oxidase | 6 | 25.4 | 5.7E-05 |
| CHK kinase-like | 9 | 11.3 | 4.4E-05 |
| Monooxygenase | 24 | 10.6 | 3.2E-15 |
| Cytochrome P450 | 21 | 9.4 | 7.4E-12 |
| Amidation | 5 | 8.9 | 2.5E-02 |
| Glutathione S-transferase | 18 | 8.7 | 1.6E-09 |
| Short-chain dehydrogenase/reductase | 10 | 8.7 | 6.9E-05 |
| UDP-glucuronosyl/UDP-glucosyltransferase | 19 | 8.6 | 6.0E-10 |
| Flavonoid biosynthetic process | 19 | 7.6 | 4.4E-09 |
| Aldolase-type TIM barrel | 8 | 7.3 | 3.6E-03 |
| Iron | 31 | 6.9 | 6.9E-15 |
| Flavoprotein | 12 | 6.4 | 5.9E-05 |
| Stress response | 13 | 6.0 | 4.4E-05 |
| HAD-like domain | 9 | 5.8 | 5.6E-03 |
| Thioredoxin-like fold | 24 | 5.1 | 2.3E-08 |
| Oxidation-reduction process | 76 | 4.1 | 5.9E-24 |
| NAD(P)-binding domain | 20 | 3.9 | 4.2E-05 |
| Protein processing in endoplasmic reticulum | 15 | 3.2 | 3.3E-03 |
| Metal-binding | 78 | 2.6 | 2.1E-12 |
| Transferase | 56 | 2.4 | 9.2E-08 |
| Zinc | 39 | 2.1 | 5.4E-04 |
| Hydrolase | 36 | 1.9 | 4.6E-03 |

**p*-value was calculated using Benjamini-Hochberg correction
