# Supplementary material for: A conserved mitochondrial surveillance pathway is required for defense against Pseudomonas aeruginosa
Source: PLoS Genet. 2017 Jun 29;13(6):e1006876. doi: 10.1371/journal.pgen.1006876 (PMC5510899; doi:10.1371/journal.pgen.1006876)
Supplement: S8 Table — (DOCX) [file pgen.1006876.s017.docx]

**Table S8. Host Response to Liquid Killing is Similar to Treatment with Phenanthroline**

| **Condition 1** | **# Genes** | **Condition 2** | **# Genes** | **Overlap** | ***p*-value** |
| --- | --- | --- | --- | --- | --- |
| Phenanthroline | 617 | PMK-1  Dependent | 101 | 4 | 0.39 |
| Liquid Killing | 167 |  |  | 2 | 0.21 |
| Phenanthroline | 617 | ZIP-2  Dependent | 25 | 11 | 10^-11^ |
| Liquid Killing | 167 |  |  | 15 | 10^-25^ |
| Phenanthroline | 617 | Hypoxia  Responsive | 110 | 34 | 10^-25^ |
| Liquid Killing | 167 |  |  | 22 | 10^-24^ |
| Phenanthroline | 617 | *S. aureus*  Infection | 189 | 16 | 0.003 |
| Liquid Killing | 167 |  |  | 4 | 0.077 |
| Phenanthroline | 617 | *Y. pestis*  Infection | 114 | 1 | 0.97 |
| Liquid Killing | 167 |  |  | 0 | 1 |
