# Supplementary material for: A conserved mitochondrial surveillance pathway is required for defense against Pseudomonas aeruginosa
Source: PLoS Genet. 2017 Jun 29;13(6):e1006876. doi: 10.1371/journal.pgen.1006876 (PMC5510899; doi:10.1371/journal.pgen.1006876)
Supplement: S9 Table — (DOCX) [file pgen.1006876.s018.docx]

**Table S9. List of Selected Human Genes with ESRE Sites.**

| Human Name | *C. elegans* Name |
| --- | --- |
| BECN1 | *bec-1* |
| C4orf33 | C33A12.3 |
| CDC42 | *cdc-42* |
| CRYAB | *hsp-16.1* |
| DUSP21 | F26A3.4 |
| GBA2 | R08F11.1 |
| GRTP1 | *tbc-6* |
| HSPA8 | F44E5.5 |
| MPI | ZK632.4 |
| PMM2 | F52B11.2 |
| UGP2 | K08E3.5 |
